# Genome-Wide Characterization of Pancreatic Adenocarcinoma Patients Using Next Generation Sequencing

**DOI:** 10.1371/journal.pone.0043192

**Published:** 2012-10-10

**Authors:** Winnie S. Liang, David W. Craig, John Carpten, Mitesh J. Borad, Michael J. Demeure, Glen J. Weiss, Tyler Izatt, Shripad Sinari, Alexis Christoforides, Jessica Aldrich, Ahmet Kurdoglu, Michael Barrett, Lori Phillips, Hollie Benson, Waibhav Tembe, Esteban Braggio, Jeffrey A. Kiefer, Christophe Legendre, Richard Posner, Galen H. Hostetter, Angela Baker, Jan B. Egan, Haiyong Han, Douglas Lake, Edward C. Stites, Ramesh K. Ramanathan, Rafael Fonseca, A. Keith Stewart, Daniel Von Hoff

**Affiliations:** 1 Translational Genomics Research Institute (TGen), Phoenix, Arizona, United States of America; 2 Mayo Clinic, Scottsdale, Arizona, United States of America; 3 Virginia G. Piper Cancer Center Clinical Trials, Scottsdale Healthcare, Scottsdale, Arizona, United States of America; 4 Arizona State University, Tempe, Arizona, United States of America; Wayne State University School of Medicine, United States of America

## Abstract

Pancreatic adenocarcinoma (PAC) is among the most lethal malignancies. While research has implicated multiple genes in disease pathogenesis, identification of therapeutic leads has been difficult and the majority of currently available therapies provide only marginal benefit. To address this issue, our goal was to genomically characterize individual PAC patients to understand the range of aberrations that are occurring in each tumor. Because our understanding of PAC tumorigenesis is limited, evaluation of separate cases may reveal aberrations, that are less common but may provide relevant information on the disease, or that may represent viable therapeutic targets for the patient. We used next generation sequencing to assess global somatic events across 3 PAC patients to characterize each patient and to identify potential targets. This study is the first to report whole genome sequencing (WGS) findings in paired tumor/normal samples collected from 3 separate PAC patients. We generated on average 132 billion mappable bases across all patients using WGS, and identified 142 somatic coding events including point mutations, insertion/deletions, and chromosomal copy number variants. We did not identify any significant somatic translocation events. We also performed RNA sequencing on 2 of these patients' tumors for which tumor RNA was available to evaluate expression changes that may be associated with somatic events, and generated over 100 million mapped reads for each patient. We further performed pathway analysis of all sequencing data to identify processes that may be the most heavily impacted from somatic and expression alterations. As expected, the KRAS signaling pathway was the most heavily impacted pathway (P<0.05), along with tumor-stroma interactions and tumor suppressive pathways. While sequencing of more patients is needed, the high resolution genomic and transcriptomic information we have acquired here provides valuable information on the molecular composition of PAC and helps to establish a foundation for improved therapeutic selection.

## Introduction

Pancreatic cancer is a malignant carcinoma that is currently the fourth leading cause of cancer-related deaths in the United States [1]. In 2011, an estimated 44,030 new patients were diagnosed, and the one- and five-year survival rates were approximately 26% and 6%, respectively [1]. Current standard treatment options for patients include surgical removal of the tumor, radiation therapy, chemotherapy, and targeted/biologic therapy. However, due to late diagnoses and the associated low survival rate, improved treatments are needed.

Significant effort by a number of groups has led to the identification of genomic alterations in pancreatic cancer. Heavily implicated genes include *KRAS* (v-Ki-ras2 Kirsten rat sarcoma viral oncogene homolog ) [2], [3], *TP53* (tumor protein p53) [4], [5], *SMAD4*/*DPC4* (SMAD family member 4/deletion target in pancreatic carcinoma 4 homolog) [6], [7], *CDKN2A* (cyclin-dependent kinase inhibitor 2A; p16) [8], [9], [10], and *BRCA2* (breast cancer 2, early onset) [11], [12]. However, FDA approved therapies that exploit these genomic alterations in pancreatic cancer are currently not available. As a result, standard agent therapy for advanced stage and metastatic pancreatic adenocarcinoma (PAC) patients commonly target tumor DNA replication, cell division, and proliferation, or specific receptors that help to mediate signaling cascades. While PAC patients commonly have mutations in the previously mentioned genes, low survival rates for PAC patients are associated with difficulty in identifying effective treatments beyond standard therapies. Such difficulty associated with finding effective treatments demonstrates that our understanding of pancreatic cancer remains limited. In order to address these challenges, one strategy is to first individually characterize patients to fully understand the range of alterations in separate tumors. In doing so, we acquire valuable information on each patient's disease, as well as PAC as a whole, and are also able to identify druggable targets that may provide additional therapeutic options on a patient-specific basis. This approach is particularly relevant because although certain mutations are common across patients, each patient's tumor demonstrates divergent aberrations. As we acquire more tumor DNA and RNA sequence information from actual patients, we will also be able to delineate the key biological processes that are central to PAC and develop improved therapies for patients.

To carry out unbiased whole genome analyses in actual patients, we performed whole genome sequencing (WGS) of tumor biopsy DNA and matched normal DNA from blood from three separate PAC patients to identify somatic events in each patient's tumor. Our primary aim is to separately characterize each of these patients to evaluate the molecular background of each tumor. To understand the possible implications of identified genomic events and to evaluate transcriptional alterations in the tumor, we also performed RNA sequencing (RNAseq) for 2 of the patients for which RNA was available. Lastly, for patients 1 and 2, we performed comparative genomic hybridization (CGH) analyses to validate copy number changes identified through sequencing. The use of next generation sequencing (NGS) and the combined analysis of separate sets of data help to create a detailed picture of the disease in each patient and contribute to our understanding of the disease. We present here very detailed genomic characterizations of three separate PAC patients.

## Materials and Methods

Detailed supplementary methods are described under Supporting Information (Methods S1). A summary of methods is presented here.

### Ethics statement

All patients were treated on protocols approved by the Mayo Clinic Institutional Review Board (MCIRB) and the Western Institutional Review Board (WIRB). This study was conducted in accordance with the 1996 Declaration of Helsinki. Written informed consent was obtained from all patients.

### Eligibility Criteria

For this study, patients had to be ≥18 years of age and provided signed informed consent. These patients included those with a pathologic or clinical diagnosis of a pancreatic malignant neoplasm, or who were undergoing a medically indicated procedure to obtain tissue or to resect their pancreatic tumor. Other eligibility criteria included: Karnofsky performance status (PS) ≥80%, life expectancy >3 months, baseline laboratory data indicating acceptable bone marrow reserve, liver, and renal function. Patients were allowed to participate on another clinical trial involving treatment prior to or during participation on this study. Main exclusion criteria included: symptomatic central nervous system (CNS) metastasis, untreated CNS metastases, known active infections requiring intravenous antimicrobial therapy, known HIV, HBV or HCV infection requiring antiviral therapy, pregnant or breast feeding women, or inaccessible tumor for biopsy.

### Sample assessment

Tumor samples were obtained under institutional review protocols and were preserved as fresh frozen. Normal DNA was obtained from peripheral blood mononuclear cells. Percent tumor cellularity of patient 1's biopsy (tumor content) was assessed as 60% tumor, patient 2 50% tumor, and patient 3 40–50% tumor. Direct visualization of samples collected from all three patients was obtained to estimate tumor content and extent of tissue heterogeneity by a board certified pathologist (GH).

### Genomic DNA isolation

Tissue was disrupted and homogenized in Buffer RLT plus (Qiagen AllPrep DNA/RNA Mini Kit), using the Bullet Blender™, Next Advance, and transferred to a microcentrifuge tube containing Buffer RLT plus and 1.6 mm stainless steel beads (patient 1), or 0.9 mm–2.0 mm RNase free stainless steel beads (patients 2 and 3). Blood leukocytes (buffy coat) were isolated from whole blood by centrifugation at room temperature and resuspended in Buffer RLT plus. All samples were homogenized, centrifuged at full speed, and lysates were transferred to the Qiagen AllPrep DNA spin column. Genomic DNA was purified following the manufacturer's protocol. DNA was quantified using the Nanodrop spectrophotometer and quality was accessed from 260/280 and 260/230 absorbance ratios.

### RNA Isolation

Tissue was disrupted and homogenized in Buffer RLT plus using the Bullet Blender, and transferred to a microcentrifuge tube containing Buffer RLT plus and 0.9 mm–2.0 mm RNAse free stainless steel beads. The tissue was homogenized in the Bullet Blender, and centrifuged at full speed. The supernatant was transferred to the QiagenAllPrep DNA spin column. 70% ethanol was added to the flow-through and the mixture was applied to an RNeasy spin column. Total RNA purification was conducted as directed by the AllPrep DNA/RNA Mini Handbook. FirstChoice normal human pancreatic RNA was purchased from Ambion (catalog#AM7954) and used as the RNAseq control. RNA was quantified using the Nanodrop spectrophotometer and quality was assessed using the Agilent Bioanalyzer.

### Whole genome library preparation

3 µg of genomic DNA from each sample was fragmented to a target size of 300–350 base pairs (bp). Overhangs in the fragmented samples were repaired and adenine bases were ligated on. Diluted paired end Illumina adapters were then ligated onto the A-tailed products. Following ligation, samples were run on a 3% TAE gel to separate products. Ligation products at 300 bp and 350 bp were selected for each sample, isolated from gel punches, and purified. 2× Phusion High-Fidelity PCR Master Mix (Finnzymes; catalog#F-531L) was used to perform PCR to enrich for these products. Enriched PCR products were run on a 2% TAE gel and extracted. Products were quantified using Agilent's High Sensitivity DNA chip (catalog#5067-4626) on the Agilent 2100 Bioanalyzer (catalog#G2939AA).

### Whole transcriptome library preparation

All RNA samples were analyzed on the Agilent Bioanalyzer RNA 6000 Nano Chip to validate RNA integrity (RIN≥7.0). 10 ng of total RNA was used to generate whole transcriptome libraries for RNA sequencing. Using the Nugen Ovation RNA-Seq System (cat#7100-08), total RNA was used to generate double stranded cDNA, which was amplified using Nugen's SPIA linear amplification process. Amplified cDNA was input into Illumina's TruSeq DNA Sample Preparation Kit – Set A (cat#FC-121-1001) for library preparation. In summary, 1 µg of amplified cDNA was fragmented to a target insert size of 300 bp and end repaired. Samples were then adenylated and indexed paired end adapters were ligated. Ligation products were run on a 2% TAE gel and size selected at 400 bp. Ligation products were isolated from gel punches and purified. Cleaned ligation products were input into PCR to enrich for libraries. PCR products were cleaned and quantified using the Agilent Bioanalyzer.

### PE next generation sequencing

Tumor and normal libraries were prepared for paired end sequencing. Clusters were generated using Illumina's cBot and HiSeq Paired End Cluster Generation Kits (catalog#PE-401-1001) and sequenced on Illumina's HiSeq 2000 using Illumina'sHiSeq Sequencing Kit (catalog#FC-401-1001).

### Array CGH (aCGH) for patient 1

Samples were run with the SurePrint G3 Human aCGH Microarray 1 M (Agilent Technologies, Palo Alto, CA). The digestion, labeling, and hybridization steps were performed as previously described with minor modifications [13]. Briefly, 1.2 ug of tumor and reference DNA were independently digested with Bovine DNase I (Ambion, Austin, TX) for 12 minutes at room temperature. DNA samples from a pool of nine human, female, lymphoblastoid cell lines from the Coriell repository (NA18517, NA19240, NA18555, NA18537, NA18980, NA18972, NA12878, NA12156, and NA15510) were used as the normal reference in the hybridization experiments. Tumor samples were labeled with Cy5 dye, and the normal reference was labeled with Cy3 dye. Labeled reactions were cleaned up and hybridized at 65°C for 40 hours. Microarrays were scanned and features were extracted with Feature Extraction software (Agilent Technologies). Log2 ratio data was analyzed using Genomic Workbench software version 5.0.14 (Agilent Technologies).

### Flow cytometry CGH for patient 2

DNA content based flow assays were used to identify and purify proliferating 2N (G1) populations, 4N(G2/M), and aneuploid populations from the biopsy. The biopsy was minced in the presence of NST buffer and DAPI according to published protocols [14], [15]. Nuclei were disaggregated immediately before analysis with a 25-gauge needle and then filtered through a 40-µm mesh filter and analyzed using an Influx cytometer (Becton-Dickinson Cytopeia, San Jose CA), with ultraviolet excitation and DAPI emission collected at >450 nm. DNA content and cell cycle were analyzed using the software program WinCycle (Phoenix Flow Systems, San Diego, CA). DNAs were extracted using Qiagen micro kits (Qiagen Valencia, CA). For hybridization, 100 ng of genomic DNA from each sample and of pooled commercial 46XX reference (Promega) were amplified using the GenomiPhi amplification kit (G.E. Healthcare, Piscataway, NJ). 1 ug of amplified sample and 1 ug of amplified reference template were digested with DNaseI and labeled with Cy-5 dUTP and Cy-3 dUTP respectively, using a BioPrime labeling kit (Invitrogen, Carlsbad, CA). All labeling reactions were assessed using a Nanodrop assay (Nanodrop, Wilmington, DE) prior to mixing and hybridization to a CGH array (Agilent Technologies, Santa Clara, CA).

### Sequencing data analysis

**Figure 1:**
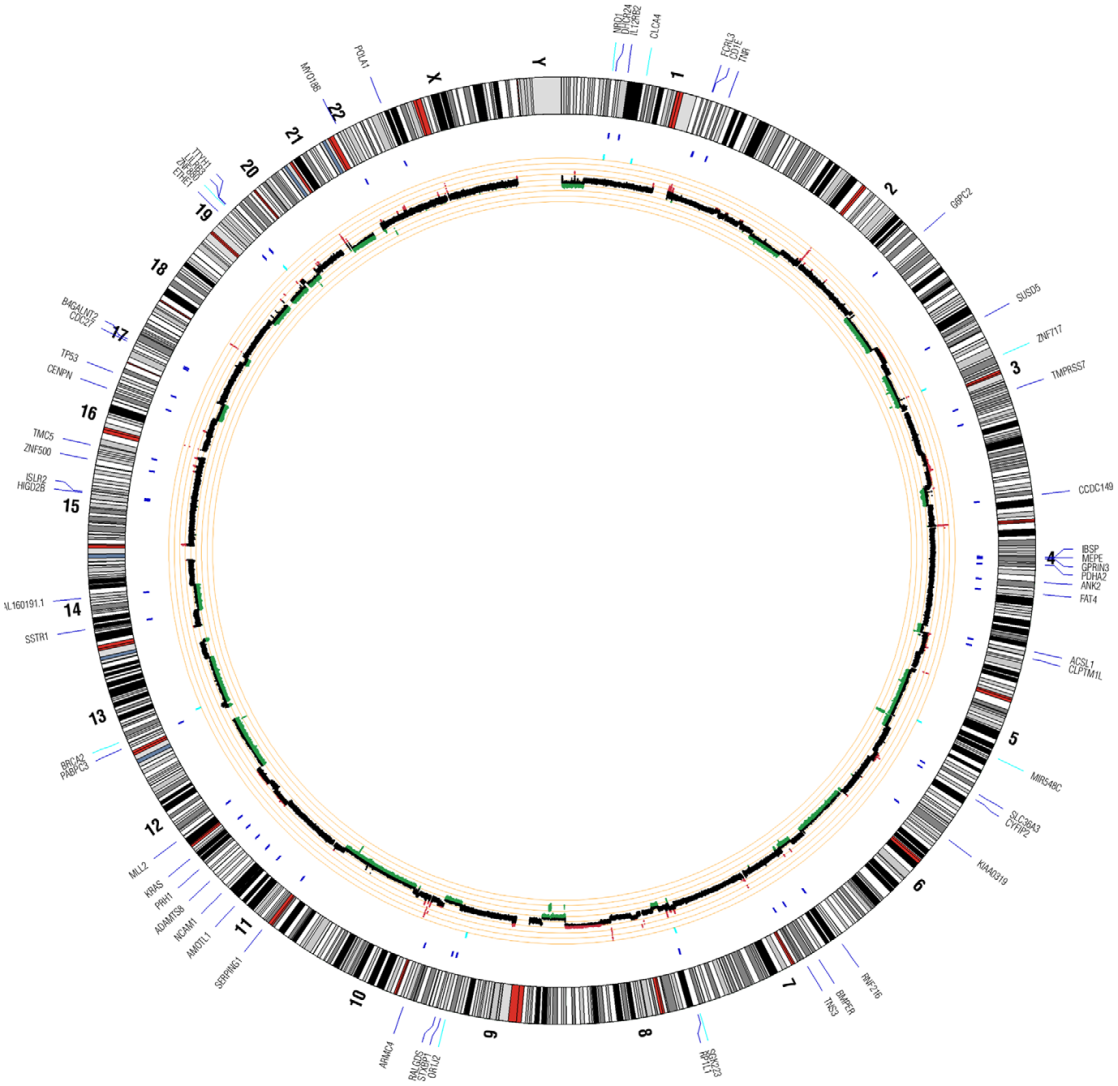
Patient 1 Circos Plot. This plot summarizes all significant genomic events that were identified in patient 1 using WGS. Copy number changes are shown in the inner circle plot with red marking amplifications and green marking deletions. SNVs are indicated with dark blue tick marks and indels are indicated with light blue tick marks.

**Figure 2:**
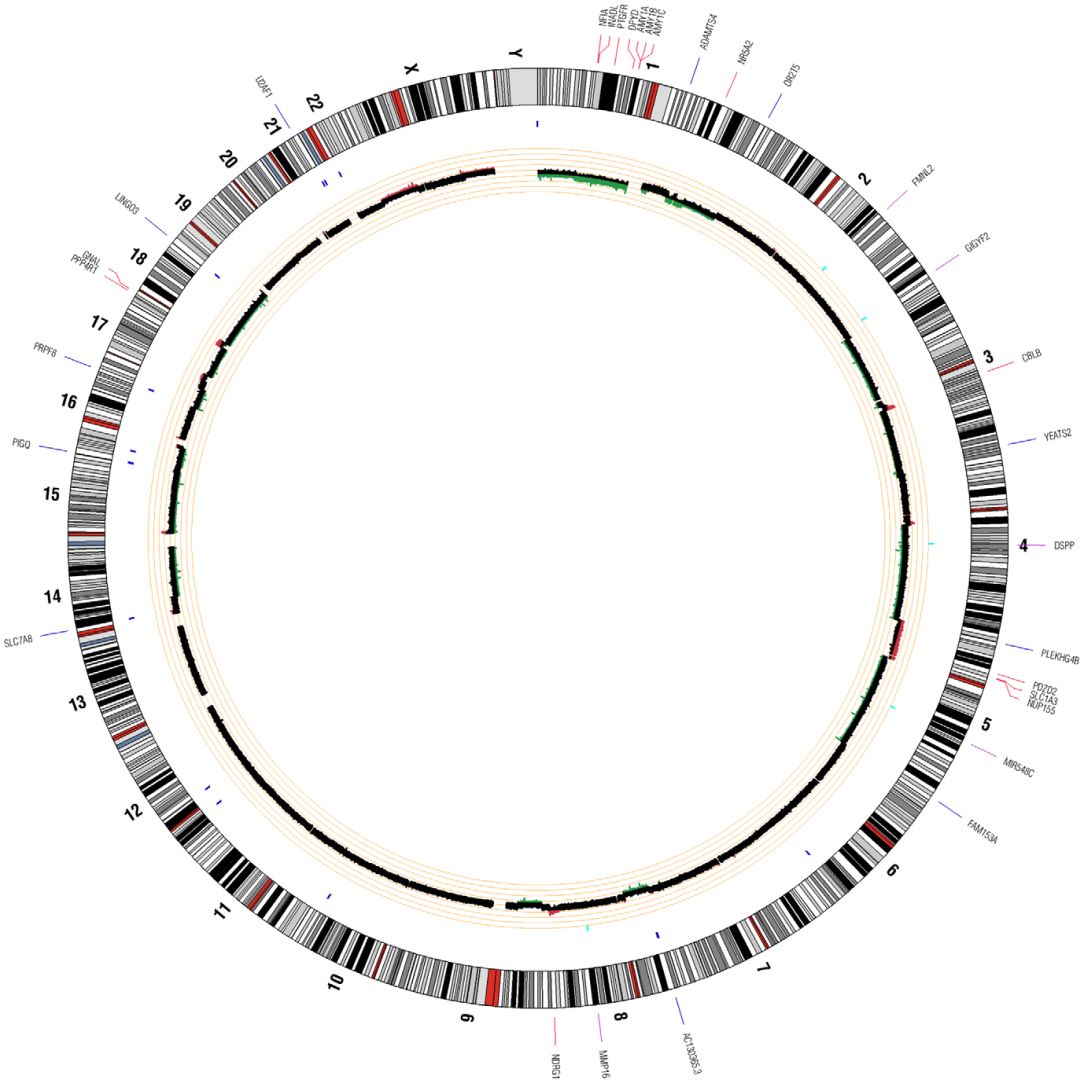
Patient 2 Circos Plot. This plot summarizes all significant genomic events that were identified in patient 2 using WGS. Copy number changes are shown in the inner circle plot with red marking amplifications and green marking deletions. SNVs are indicated with dark blue tick marks and indels are indicated with light blue tick marks.

**Figure 3:**
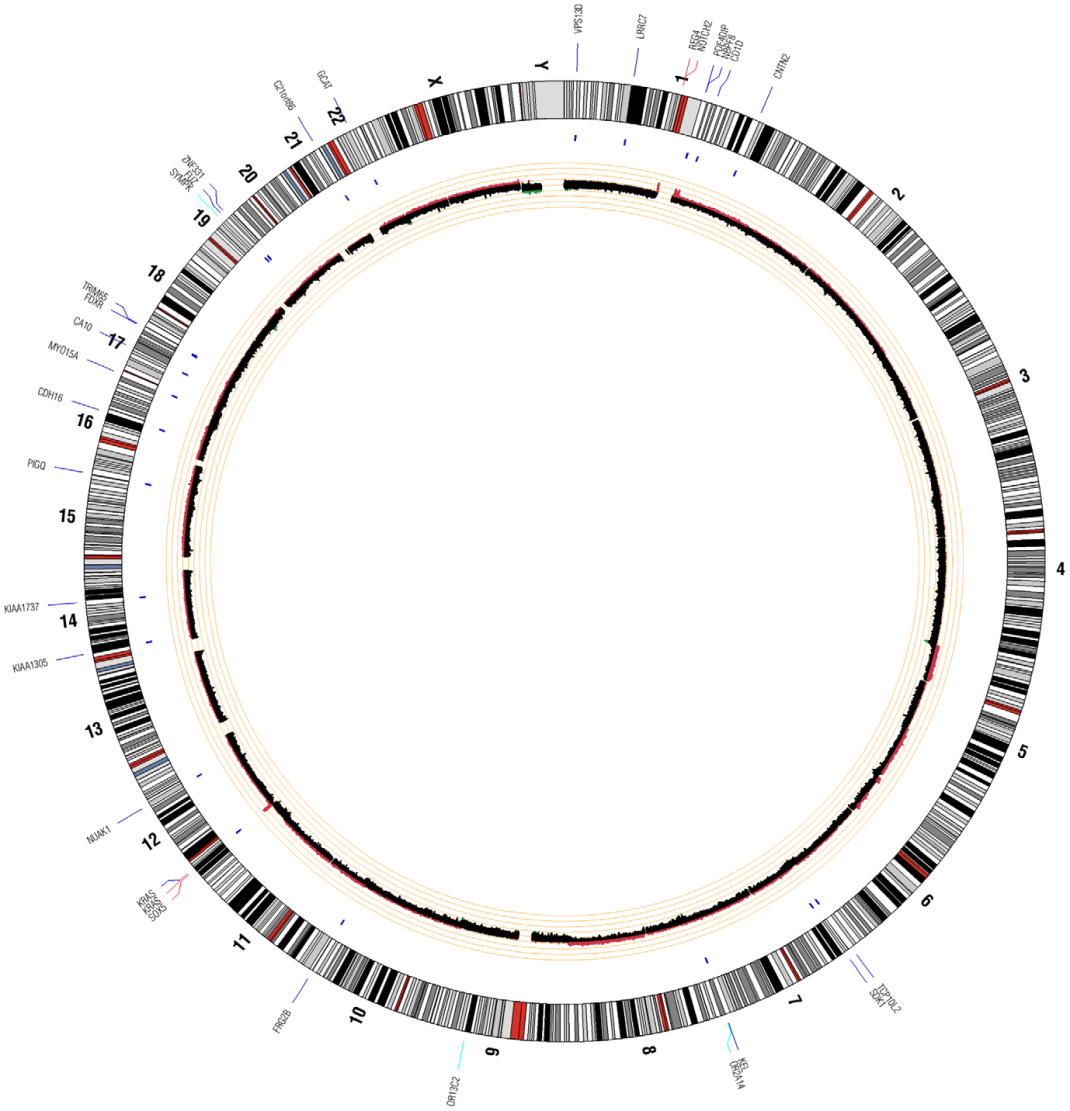
Patient 3 Circos Plot. This plot summarizes all significant genomic events that were identified in patient 3 using WGS. Copy number changes are shown in the inner circle plot with red marking amplifications and green marking deletions. SNVs are indicated with dark blue tick marks and indels are indicated with light blue tick marks.

Raw sequence data in the form of .bcl files were generated by the Illumina HiSeq 2000. These data were converted to .qseq files, which were used to generate .fastq files. Fastq files were validated to evaluate the distribution of quality scores and to ensure that quality scores do not drastically drop over each read. Validated fastq files were aligned to the human reference genome (build 36) using the Burrows-Wheeler Alignment (BWA) tool. Following alignment,.sai files were used to create .sam (sequence alignment map) files [16], which were input into SAMtools to create binary sequence (.bam) files. PCR duplicates were flagged for removal using Picard. Indels were realigned and base quality scores were recalibrated using GATK (Genome Analysis Toolkit) [17]. Mutation analysis was performed to identify SNPs, indels, and CNVs. Circos plots were generated for each patient to summarize results from all variant analyses (Figures 1, 2, and 3). NCBI (National Center for Biotechnology Information) SRA (Sequence Read Archive) accession numbers for each patient are as follows—patient 1 DNA: SRS334038, SRS334039; patient 2 DNA: SRS334040, SRS334041; patient 2 RNA: SRS348787; patient 3 DNA: SRS334042, SRS334045; patient 3 RNA: SRS348788; pancreas RNA control: SRS334047).

SNP (single nucleotide polymorphism) calling was performed using SolSNP (http://sourceforge.net/projects/solsnp/) and Mutation Walker, a tool developed in house and that incorporates variant discovery tools from GATK. SNPs that were called using both tools were compiled and visually examined for false positives to create a final filtered list of true SNVs (single nucleotide variants). Indel (insertion/deletion) calling was performed using GATK and a somatic indel detection tool developed in house. SIFT (Sorting Intolerant From Tolerant) or PolyPhen-2 (Polymorphism Phenotyping v2) was used to determine the effect of coding SNV's and indels on protein function. Copy number analysis was completed by determining the log2 difference of the normalized physical coverage (or clonal coverage) for both germline and tumor samples separately across a sliding 2 kb window of the mean. CREST (Clipping Reveals Structure) was used on WGS data to identify structural variations [18].

RNAseq data was aligned against human reference genome (build 36) with TopHat 1.2; RNAseq reads were only aligned against the autosomes and sex chromosomes. Mitochondrial DNA and annotations were removed from the genome and annotation references prior to alignment. Cuffdiff was used to identify differentially expressed genes and isoforms. Differential analysis was performed on FPKM (Fragments Per Kilobase of transcript per Million fragments mapped) expression values calculated for gene and isoform. P-values were corrected for multiple testing using the Benjamini and Hochberg method. ChimeraScan [19] was used for fusion transcript detection.

#### Pathway analysis

Integrative analysis of whole genome and transcriptomic data was performed using the Functional Ontology Enrichment Tool in MetaCore from GeneGo, Inc. (v6.8; Thomson Reuters Business, Philadelphia, PA). Pathway analysis specific to pancreatic cancer was performed using the MetaMiner (Oncology) Pancreatic Cancer Disease Module add-on. P-values associated with each analysis are calculated in MetaCore using a hypergeometric distribution.

## Results and Discussion

### Whole genome sequencing

Our study was performed on a set of fresh pancreatic tumor specimens and whole blood samples from three patients diagnosed with PAC. Clinical information is listed in Table 1. For each patient, we sequenced both tumor DNA, as well as germline DNA isolated from whole blood in order to identify somatic changes in the tumors. Read alignment was performed with BWA using build 36 of the human reference genome. WGS metrics and summary statistics for each of the three patients are shown in Table 2. Using sequencing by synthesis technology and 100 bp paired end chemistry, we generated nearly 8 billion total reads from WGS for average mapped coverages ranging from 31× to 54×. SNP calling was performed using two separate callers to reduce the false negative rate. To evaluate the overall quality of variant data, germline SNPs were called and the transition to transversion and dbSNP (Single Nucleotide Polymorphism Database) [20] 129 concordance ratios were calculated. For all three patients, the transition/transversion ratios were in the range of 2.01 to 2.24, and the dbSNP 129 concordance ratios were approximately 87% (Table 2). These analyses indicate that no biases were encountered with respect to nucleotide substitutions, that SNPs identified in the data strongly correlate with common genetic variations, and that high quality variant calling was performed.

**Table 1:** Patient clinical information.

	Patient 1	Patient 2	Patient 3
Age at diagnosis (years)	55	76	57
Gender	male	female	male
Ethnicity	Caucasian	Caucasian	Caucasian
Diagnosis	adenocarcinoma w/liver metastases	adenocarcinoma w/no metastasis	adenocarcinoma w/liver metastases
Tumor stage	IV	IIB	IV
Tumor grade	poorly differentiated	moderately differentiated	poorly differentiated
Tumor content	60%	50%	40–50%
Sequenced biopsy	liver metastasis	primary tumor	liver metastasis
Clinical status	Received treatment^a^: deceased	Did not receive treatment: no recurrence after 24 months	Received treatment^b^: deceased

a Clinical benefit with FOLFOX (folinic acid, fluorouracil, oxaliplatin) systemic therapy for 24 weeks with 98% maximal serum CA19-9 reduction and partial metabolic response by EORTC PET criteria.

b Transient clinical benefit with FOLFOX systemic therapy for 10 weeks with maximal serum CA19-9 reduction of 36% and RECIST (Response Evaluation Criteria in Solid Tumors) reduction of 21% in sum of largest diameters.

**Table 2:** WGS and RNAseq metrics.

WGS metrics		Patient 1	Patient 2	Patient 3	Normal human pancreas
	Total amount of data generated (GB)	271.75	315.80	420.3	-
	Q30 data generated (GB)	207.80	237.67	352.7	-
	Average Total cluster densities (K/mm^2^)	381.36	681.31	569.21	-
	Average PF cluster densities (K/mm^2^)	348.80	521.76	466.84	-
	Average PF rate	76.43	76.61	83.4	-
	Total number of reads	2256848363	2767484751	2878046795	-
	Aligned Reads - Normal	1052366015	1441444310	1271057635	-
	Aligned Reads - Tumor	1204482348	1326040441	1606989160	-
	Aligned Bases - Normal	96863052455	1.4991E+11	1.3219E+11	-
	Aligned Bases - Tumor	1.11483E+11	1.37908E+11	1.67127E+11	-
	Average coverage depth - Normal	31.31	48.46	42.73	-
	Average coverage depth - Tumor	36.04	44.58	54.03	-
	Variant Analysis	BWA	BWA	BWA	-
	Germline SNPs called	2013281	2129857	3610297	-
	Transition/Transversion Ratio	2.24	2.17	2.01	-
	dbSNP 129 rate	87.59	87.65	87.29	-
	Non-synonymous germline variants	504	10151	12830	-
	Somatic SNVs called (strict lists)	20323	714	25	-
	False Positives (in dbSNP or 1000 Genomes) (strict lists)	0.107	0.41	0.36	-
	Somatic indels called (CODING and UTR)	8	5	3	-
**RNAseq metrics**					
	Total amount of data generated (GB)	-	25.7	22.4	14.8
	Q30 data generated (GB)	-	21.1	18.4	12.2
	Average Total cluster densities (K/mm^2^)	-	810.0	694.0	1063.0
	Average PF cluster densities (K/mm^2^)	-	680.4	589.2	533.6
	Average PF rate	-	84.0	84.9	50.2
	Total number of reads	-	272694175	247382440	377376444
	Total mapped reads	-	124914613	104693716	98290756

#### Variant Analysis

Aligned reads for both tumor and normal libraries were evaluated to identify genomic events including non-synonymous SNVs (nsSNVs), indels, and copy number variants (CNVs). Summaries of identified variants in each patient are shown in Figures 1, 2, and 3. Across all 3 patients, 142 coding genomic events were identified. A total of 101 events were identified in patient 1, 17 in patient 2, and 24 in patient 3. Of these events, we identified 11 indels (Table 3), 69 nsSNVs (Table 3), and 62 focal/chromosomal CNVs (Table 4). These 62 CNVs encompass approximately 4,576 genes across all 3 patients. 119 COSMIC (Catalogue of Somatic Mutations in Cancer) genes that fall within these CNV regions are listed in Table 4. Using CREST, we did not identify any significant somatic structural variants in the 3 patients.

**Table 3:** Indels and SNVs identified through WGS.

Patient	Chr.	Location	Gene Name	Coding event	Alteration	Sequence Change	Effect^a^
1	13	31805365	*BRCA2*	Indel	deletion	AAAAG	NMD^b^; frameshift
1	1	86818484	*CLCA4*	Indel	deletion	CCTACA	no NMD
1	1	52078651	*NRD1*	Indel	deletion	TCT	no NMD
1	9	124313206	*OR1J2*	Indel	insertion	T	NMD unknown; frameshift
1	8	8272117	*SGK223*	Indel	insertion	G	NMD; frameshift
1	19	57578957	*ZNF880*	Indel	deletion	A	NMD; frameshift
1	17	7518264	*TP53*	SNV	R248W	G/A	damaging
1	12	25289551	*KRAS*	SNV	G12V	C/A	damaging
1	4	55642955	*KDR*	SNV	T1258M	G/A	damaging
1	3	131766828	*COL6A6*	SNV	S321N	G/A	tolerated
1	4	185938530	*ACSL1*	SNV	K143X	T/A	termination
1	11	129794256	*ADAMTS8*	SNV	L288F	G/A	damaging
1	11	94172689	*AMOTL1*	SNV	R229X	C/T	termination
1	4	114415228	*ANK2*	SNV	G553R	G/A	damaging
1	10	28312788	*ARMC4*	SNV	F270Y	A/T	tolerated
1	17	44591549	*B4GALNT2*	SNV	T217M	C/T	damaging
1	7	33976531	*BMPER*	SNV	W123X	G/A	termination
1	4	24419470	*CCDC149*	SNV	A336G	G/C	damaging
1	17	42569603	*CDC27*	SNV	H615Q	A/T	damaging
1	16	79619379	*CENPN*	SNV	A305P	G/C	damaging
1	5	1387414	*CLPTM1L*	SNV	A294V	G/A	damaging
1	5	156718707	*CYFIP2*	SNV	R232M	G/T	tolerated
1	1	55090513	*DHCR24*	SNV	C511F	C/A	damaging
1	11	117156429	*DSCAML1*	SNV	R118H	C/T	tolerated
1	19	48702891	*ETHE1*	SNV	F239S	A/G	tolerated
1	4	126592198	*FAT4*	SNV	L1824S	T/C	tolerated
1	2	169467273	*G6PC2*	SNV	C97X	C/A	termination
1	4	144580767	*GAB1*	SNV	P456Q	C/A	damaging
1	4	90388090	*GPRIN3*	SNV	R732L	C/A	damaging
1	1	67628381	*IL12RB2*	SNV	K676N	G/T	damaging
1	15	72213782	*ISLR2*	SNV	R545H	G/A	damaging
1	X	48707520	*KCND1*	SNV	R158H	C/T	damaging
1	6	24664901	*KIAA0319*	SNV	D924N	C/T	tolerated
1	19	59437727	*LILRA6*	SNV	E114D	C/A	tolerated
1	4	88985483	*MEPE*	SNV	I147F	A/T	damaging
1	7	141354973	*MGAM*	SNV	K109M	A/T	damaging
1	12	47726693	*MLL2*	SNV	L1462F	G/A	no prediction
1	22	24494160	*MYO18B*	SNV	D95H	G/C	tolerated
1	11	112610991	*NCAM1*	SNV	V8M	G/A	tolerated
1	4	96980836	*PDHA2*	SNV	L171P	T/C	damaging
1	X	24816075	*POLA1*	SNV	R1360H	G/A	tolerated
1	12	10926455	*PRH1*	SNV	Q70H	C/A	damaging
1	9	134965563	*RALGDS*	SNV	V773I	C/T	damaging
1	7	5658630	*RNF216*	SNV	H643P	T/G	tolerated
1	8	10505716	*RP1L1*	SNV	P1101H	G/T	damaging
1	11	57138427	*SERPING1*	SNV	P477T	C/A	tolerated
1	4	975236	*SLC26A1*	SNV	Q86K	G/T	damaging
1	5	150648782	*SLC36A3*	SNV	E89X	C/A	termination
1	14	37748706	*SSTR1*	SNV	R121C	C/T	tolerated
1	16	1068870	*SSTR5*	SNV	M1V	A/G	damaging
1	9	129482338	*STXBP1*	SNV	V515I	G/A	tolerated
1	3	33170461	*SUSD5*	SNV	L223V	G/C	tolerated
1	16	19359304	*TMC5*	SNV	G148V	G/T	damaging
1	3	113263420	*TMPRSS7*	SNV	K343N	G/T	tolerated
1	1	173638901	*TNR*	SNV	A325E	G/T	tolerated
1	19	59634093	*TTYH1*	SNV	P346L	C/T	tolerated
1	18	72721138	*ZNF236*	SNV	V354L	G/T	tolerated
1	16	4755940	*ZNF500*	SNV	E14G	T/C	tolerated
2	4	88756318	*DSPP*	Indel	deletion	GACAGCAGC	no NMD; frameshift
2	2	153184312	*FMNL2*	Indel	insertion	CCA	no NMD
2	2	233420470	*GIGYF2*	Indel	deletion	ACA	NMD; frameshift
2	8	89150850	*MMP16*	Indel	insertion	A	NMD unknown; frameshift
2	12	25289551	*KRAS*	SNV	G12V	C/A	damaging
2	19	2242562	*LINGO3*	SNV	G72S	C/T	tolerated
2	17	1508349	*PRPF8*	SNV	F1818C	A/C	damaging
2	21	43397525	*U2AF1*	SNV	S34F	G/A	damaging
3	19	51042923	*SYMPK*	Indel	deletion	GA	no NMD
3	12	25289552	*KRAS*	SNV	G12R	C/G	damaging
3	17	47065916	*CA10*	SNV	R295H	C/T	damaging
3	1	156418469	*CD1D*	SNV	A118T	G/A	tolerated
3	16	65505678	*CDH16*	SNV	L241P	A/G	tolerated
3	10	135290149	*FRG2B*	SNV	T30S	T/A	tolerated
3	19	55006332	*FUZ*	SNV	L198F	G/A	possibly damaging
3	7	142361153	*KEL*	SNV	A313T	C/T	tolerated
3	14	23954072	*KIAA1305*	SNV	A1093T	G/A	damaging
3	14	76650235	*KIAA1737*	SNV	R341C	C/T	damaging
3	1	70277766	*LRRC7*	SNV	A1191V	C/T	damaging
3	7	4251904	*SDK1*	SNV	A2108T	G/A	tolerated
3	1	12300948	*VPS13D*	SNV	E2461K	G/A	tolerated
3	19	58772525	*ZNF331*	SNV	E300A	A/C	damaging

a Effects were determined using SIFT/Polyphen-2.

b NMD = nonsense mediated decay.

**Table 4:** Copy number changes identified through WGS.

Patient	Chromosome	CNV^a^	Physical Position (Mb)	Patient	Chromosome	CNV^1^	Physical Position (Mb)
1	1p	Loss	0.8–29.0	1	13q	Focal Loss	18.6–20.7
1	1q	Focal Gain	143.7–144.0	1	13q	Loss	25.2–87.2
1	20p	Loss	0.2–18.8	1	13q	Focal Loss	111.7–114.2
1	21p	Focal Gain	9.9	1	14q	Loss	41.4–73.3
1	21q	Loss	13.9–46.9	1	15q	Focal Gain	19.3
1	22q	Focal Loss	15.4–16.7	1	16p	Focal Loss	0.5–1.3
1	2p	Loss	17.6–63.3	1	16q	Focal Gain	69.7
1	2q	Loss	189.0–242.5	1	17p	Loss	0.06–21.2
1	3p	Loss	38.5–77.2	1	18p	Loss	3.2–10.7
1	3q	Gain	162.1–175.5	1	18q	Focal Loss	71.0–76.0
1	4p	Loss	0.3–20.7	1	19p	Loss	0.2–24.1
1	4q	Loss	184.0–189.4	1	19q	Loss	34.3–59.4
1	5q	Loss	52.9–133.8	2	1p	Loss	53.3–115.0
1	5q	Focal Loss	69.3–70.4	2	1q	Loss	177.8–198.4
1	5q	Focal Loss	118.3–119.0	2	3q	Focal Gain	106.7–107.0
1	6p	Focal Loss	32.1–32.1	2	5p	Focal Gain	1.3
1	6q	Loss	57.1–134.6	2	5p	Gain	31.5–50.8
1	6q	Loss	154.4–170.8	2	8q	Focal Gain	131.2–135.7
1	6q	Focal Loss	157.6–158.0	2	15q	Focal Gain	19.8–19.9
1	6q	Focal Loss	167.9–168.0	2	17p	Focal Loss	0.09
1	7p	Loss	0.5–6.0	2	18p	Gain	9.1–14.2
1	7q	Focal Loss	74.1	3	1p	Focal Loss	1.1–3.6
1	8p	Focal Loss	21.9–30.1	3	1p/q	Gain	120.0–143.7
1	8q	Gain	100.8–146.3	3	3q	Focal Loss	121.8–121.9
1	9p	Loss	0.3–27.5	3	4p	Focal Loss	1.7–3.4
1	9p	Focal Loss	19.7–22.0	3	4q	Focal Loss	69.1
1	10p	Loss	0.2–22.4	3	5p	Focal Gain	32.4
1	10q	Loss	67.6–135.3	3	9q	Focal Loss	136.3–138.4
1	11p	Loss	0.2–36.3	3	12p	Focal Gain	23.9–26.4
1	12q	Loss	60.5–132.3	3	18q	Focal Loss	74.8–75.3

a Focal gains/losses are defined as CNVs occurring across regions that are < = 5 Mb.

### Whole transcriptome sequencing

Whole transcriptome sequencing was performed for patients 2 and 3 and normal human pancreatic RNA (Materials and Methods). RNAseq was not performed for patient 1 because tumor RNA was not available. An average of 109 million mapped reads was generated across the 3 analyzed samples. Tumor RNAseq data was compared to normal human pancreatic RNAseq data to identify expression changes in the tumor biopsies. Whole transcriptome sequencing metrics are listed in Table 2.

#### RNA-seq Analysis

Overall, in patient 2, 1,841 genes showed significant expression changes (q<0.05, corrected for multiple testing), whereas in patient 3, 1,939 genes showed significant changes. From these two analyses, 877 common genes/isoforms were identified as showing significant expression changes. Genes demonstrating both CNVs and significant expression changes (in patients 2 and 3) are listed in Table S1. Putative fusion transcripts identified in patients 2 and 3 are listed in Table S2.

### Patient 1 analysis

#### Whole genome analysis

Well-established genes implicated in PAC include *BRCA2*, *TP53*, *CDKN2A* (p16), *MYC* (v-myc myelocytomatosis viral oncogene homolog), *SMAD4*, and *KRAS*. Compared to the other sequenced patients, patient 1 harbored the majority of genomic events in these genes including a deletion within and CNV loss encompassing *BRCA2*, an SNV in *TP53* (a nonsynonymous mutation along with 17p hemizygous loss of the wildtype allele), a homozygous deletion of the *CDKN2A* locus, and an interstitial 8q CNV gain encompassing *MYC*. The deletion identified in *BRCA2* in patient 1 causes a frameshift and nonsense-mediated decay of the transcript, whereas SNVs identified in *TP53*, *KRAS*, and *KDR* are all associated with damaging effects on the coding product. The alterations that affect *BRCA2* suggest that DNA repair mechanisms may be affected, thereby, providing an explanation for the high number of somatic aberrations identified in patient 1 compared to the other sequenced patient tumors. *BRCA2* germline mutations, in addition to being associated with increased risk of breast and ovarian cancers [21], [22], [23] also occurs in a small subset of both familial pancreatic cancer cases [24], [25]. Although *BRCA2* mutations have been identified in PAC, the deletion we identify here in exon 10 of *BRCA2* has not been previously reported.

The R248 SNV identified in *TP53* has been previously reported in multiple cancers [26], [27]. *TP53* also fell within a region of CNV loss in patient 1. The missense mutation is predicted to be damaging and the SNV and CNV loss suggest that tumor suppressor activity of TP53 may be compromised. Furthermore, *MDM2* (Mdm p53 binding protein homolog) demonstrated a CNV loss. MDM2 is involved in regulation of TP53 activity such that the cumulative effect of its CNV loss, along with the alterations identified in *TP53*, suggest that regulation of TP53 and TP53's normal functions are impacted. A homozygous deletion of *CDKN2A* was also identified to indicate that p16 tumor suppressor functions are likely compromised. CNV loss of *CDKN2A* has been previously reported in PAC [28]. Patient 1 also demonstrated a previously reported mutation in *KRAS* for which glycine (G) is converted to valine (V) at amino acid position 12 [29], [30], [31]. The SNV in *KDR*, which codes for a tyrosine kinase VEGF (vascular endothelial growth factor) receptor, has not been previously reported in PAC. These genomic events identified in *KDR* and *KRAS* may lead to dysregulation of signaling cascades upstream of tumor cell proliferation to help promote tumor growth.

Copy number gains encompassing *MYC* indicate that this gene is likely oncogenic in patient 1. Amplification of *MYC* has been reported in PAC [32], [33], and one study identified a positive correlation between *MYC* amplification and tumor grade but not survival [34]. Aside from commonly reported genes in PAC, *APC* (adenomatous polyposis coli), *MAP2K4* (mitogen-activated protein kinase kinase 4), *FHIT* (fragile histidine triad), and *AKT2* (v-akt murine thymoma viral oncogene homolog 2) also fell in regions of CNV loss. Mutations in *APC* have been reported in PAC [35], [36], [37], and *APC* copy number loss has been reported in colorectal cancer [38], [39] and gastric cancer [40]. Due to APC's function as a tumor suppressor, decreased copy number of this gene in patient 1 likely represents a key inactivating event in patient 1's cancer. Similar to *APC*, *FHIT* and *MAP2K4*, which both may act as tumor suppressors [41], , demonstrated copy number losses and may also represent inactivating aberrations. A copy number loss in *FHIT* has also been previously reported in PAC [28], and mutations in *MAP2K4* have been identified in pancreatic and other cancers [41], [42]. Lastly, *AKT2*, a putative oncogene, has been reported to be amplified in pancreatic cancer [44].

Somatic CNV losses identified using WGS also encompassed *RB1* (retinoblastoma 1), another tumor suppressor. Copy number losses in *TP53*, *AKT2*, *APC*, *MAP2K4*, and *RB1* represent key events likely associated with tumor progression and growth in patient 1. Additional relevant genes that fell in CNV regions identified using WGS are listed in Table 4 and include *PIK3R1* (phosphoinositide-3-kinase, regulatory subunit 1 (alpha)), *MLLT3* (myeloid/lymphoid or mixed-lineage leukemia), *FGFR2* (fibroblast growth factor receptor 2), *ALK* (anaplastic lymphoma receptor tyrosine kinase), *EML4* (echinoderm microtubule associated protein like 4), and *HRAS* (v-Ha-ras Harvey rat sarcoma viral oncogene homolog), all of which demonstrated copy number losses and all of which have not been reported in PAC. A total of 43 regions demonstrating CNV alterations, and which encompassed 4,426 genes, were identified in patient 1. Structural variant analysis did not identify any aberrations in the tumor genome of patient 1.

We further performed aCGH analysis on patient 1's tumor and validated all CNVs described here (Table S3). aCGH analysis also identified biallelic deletion of *NF2* (neurofibromin 2), a tumor suppressor gene, which was initially not reported due to CNV threshold cutoffs in the WGS analysis but which was subsequently confirmed in the whole genome sequence data. This gene has not been implicated in PAC but one study on pancreatic endocrine tumors localized tumor suppressor loci to regions that include *NF2* [45].

#### Summary

Many patients who are treated with gemcitabine and 5-FU based treatments often fail and are thus interested in and positioned to try additional agents that might offer benefit. Knowledge of the specific mutations in a patient's cancer may indicate targetable drivers and an oncologist and physician may decide to empirically treat the tumor based off the hypothesis that targeting the mutant may offer benefit. Our WGS findings thus provide insight into potential therapeutic options as well as patients' responses to treatments. For patient 1, based off the deletion and copy number loss identified for *BRCA2*, potential therapies include platinum compounds (cisplatin/carboplatin), mitomycin C, or alkylators. Following the collection of the tumor biopsy for sequencing, patient 1 was treated with a platinum compound (oxaliplatin) as a part of FOLFOX (folinic acid, fluorouracil, oxaliplatin) treatment. Patient 1 showed a complete response, but subsequently developed resistance 6 months later. Furthermore, the copy number loss identified for *AKT2* may be associated with patient 1's initial response to gemcitabine prior to biopsy as a recent study showed that AKT2 inhibition is associated with increased gemcitabine sensitivity [46]. Other studies also show that inhibition or silencing of AKT2 may block the growth of tumor cells and tumor formation [47], [48]. Patient 1's partial response was measured by EORTC PET (European Organisation for Research and Treatment of Cancer positron emission tomography) criteria along with normalization of CA19-9 (after six months, the cancer progressed and developed elevation in CA19-9). Overall, the *BRCA2* deletion is likely the driving mutation in this patient as the loss of DNA repair functions permits the occurrence of mutations that, in this patient, affected numerous genes including tumor suppressors. Given this finding, the use of PARP (poly ADP ribose polymerase) inhibitors may have represented a viable therapeutic option. Lowery *et al.* reported treatments and responses of pancreatic cancer patients with *BRCA* mutations and demonstrated the utility of using PARP (poly (ADP-ribose) polymerase) inhibitors for these patients. This finding and association provides evidence of the utility of performing whole genome analyses of patients in order to identify less common mutations that may be relevant for therapeutic selection. Our identification of copy number losses in *EML4* and *ALK*, as well as the absence of an *EML4-ALK* fusion, also provides evidence that crizotinib, an ALK inhibitor typically used to treat non-small cell lung cancer, would not be an option for this patient. Lastly, potential therapies that may be considered based on the *KDR* mutation include sunitinib, a tyrosine kinase inhibitor, and bevacizumab, which blocks the action of VEGFA (vascular endothelial growth factor A).

### Patient 2 analysis

#### Whole genome analysis

Patient 2 did not harbor any events in *BRCA2*, *TP53*, *CDKN2A*, *SMAD4*, or *MYC*. Like patient 1, patient 2 also demonstrated a mutation in *KRAS* at the same position (G12V). Overall, patient 2 demonstrated much fewer genomic aberrations compared to patient 1 and did not demonstrate aberrations affecting DNA repair genes. Structural variant analysis using CREST did not identify any significant somatic events in patient 2.

Aside from *U2AF1* (U2 small nuclear RNA auxiliary factor 1), SNVs and indels identified in patient 2 affect genes that have not been previously reported in PAC or COSMIC. U2AF1 functions as a part of the spliceosome and mutations in this gene have been identified in myeloid hematopoietic cancers including chronic myelomonocytic leukemia [49], [50]. The SNV in *U2AF1* identified in patient 2 is predicted to be damaging such that proper splicing of transcripts may be affected. A nine base pair deletion, causing a frameshift, was identified in *DSPP* (dentin sialophosphoprotein), which has been reported in oral squamous cell carcinoma [51].This gene codes for tooth extracellular matrix proteins so its potential role in PAC is unclear. We identified a frameshift insertion in *FMNL2* (formin-like 2), which normally functions to regulate processes requiring actin, including cytokinesis, invasion, and cell motility. Although *FMNL2* mutations have not been reported in PAC, it may have roles in colorectal carcinoma [52], [53], [54] and hepatocellular carcinoma [55]. For *GIGYF2* (GRB10 interacting GYF protein 2), we identified a frameshift deletion. GIGYF2 was shown to interact with RQCD1 (RCD1 required for cell differentiation1 homolog) and may be involved in regulating the activity of AKT in the EGFR pathway in breast cancer [56],[57]. Although *GIGYF2* has not been described in PAC, the deletion and resulting frameshift in patient 2 may affect normal functions associated with AKT regulation. Interestingly, *MMP16* (matrix metallopeptidase 16 (membrane-inserted)), which shows a single base insertion in patient 2, was previously found to be the target of a micro-RNA whose over-expression inhibited migration and invasion of the MIA PaCa-2 pancreatic cancer cell line [58]. This finding suggests that MMP16 may be involved with migration and invasion of pancreatic cancer cells. In a recent exome sequencing study of intraductal papillary mucinous neoplasms of the pancreas, *PRPF8* (PRP8 pre-mRNA processing factor 8 homolog) was recently found to garner a mutation (A1842V) resulting from a SNV (C>T) [59]. This mutation differs from the SNV we identified in patient 2, and has not been reported in PAC or other cancers, but provides evidence of a potential role of this gene, which functions in pre-mRNA splicing, in PAC.

In patient 2, we identified 9 regions, covering 114 genes that demonstrate copy number alterations (Table 4). These regions encompass *CBLB* (Cbl proto-oncogene, E3 ubiquitin protein ligase B), *IL7R* (interleukin 7 receptor), *LIFR* (leukemia inhibitory factor receptor alpha), and *NDRG1* (N-myc downstream regulated 1), all of which showed copy number gains. *CBLB* has not been implicated in PAC, but mutations in this gene have been identified in leukemias [60], [61]. *IL7R* also has not been previously reported in PAC, but has been found to demonstrate activating mutations in lymphoblastic leukemias [62], [63], [64]. *LIFR* has been reported in other malignancies including colorectal and hepatocellular carcinomas [65], [66], and is also suggested to have a role in tumor growth in pancreatic cancer [67]. Lastly, *NDRG1* has not been reported in pancreatic cancer but is suggested to arrest metastasis in prostate and colon cancers [68], [69]and it was also shown that *NDRG1* expression suppresses tumor cell growth [70].

Copy number validation was performed using flow sorted aCGH which involves flow sorting nuclei from the tumor biopsy to identify aneuploid populations. The sorted aneuploid population is then separately analyzed using aCGH. Using this analysis, we validated CNV gains identified using WGS in *CBLB*, *IL7R*, *LIFR*, and *NDRG1* (Table S3).

#### Whole transcriptome analysis

1,841 genes demonstrating significant expression changes (q<0.05, corrected for multiple testing) in the tumor were identified. COSMIC genes demonstrating significant expression changes are listed in Table 5. Genes showing significantly altered expression in the tumor and that also fall in regions of copy number change are listed in Table S1. Putative fusion transcripts identified in patient 2, of which 2 contributing genes showed significantly altered expression, are listed in Table S2. As structural variant analysis did not detect significant somatic aberrations, the fusion transcripts detected in patient 2 are not correlated with genomic data.

**Table 5:** Selected^a^ differentially expressed genes identified using RNAseq^b^.

Patient	Gene	ln (fold change)	q-value (corrected)	Patient	Gene	ln (fold change)	q-value (corrected)
2	*AKT3*	−4.37	2.92E-03	3	*ABL1*	5.51	5.50E-03
2	*ATM*	−3.84	2.25E-02	3	*AKT2*	4.42	4.52E-02
2	*ATRX*	−3.19	4.89E-02	3	*ATRX*	−5.32	3.12E-05
2	*ATRX*	−5.68	5.53E-06	3	*BCL3*	4.43	4.40E-03
2	*BCL3*	4.42	4.77E-03	3	*BCL3*	3.93	1.40E-02
2	*BCL3*	4.25	1.05E-02	3	*BIRC3*	6.64	2.06E-07
2	*BIRC3*	5.86	1.67E-05	3	*BRCA1*	5.22	2.58E-04
2	*BRCA2*	3.48	2.31E-02	3	*CDH1*	−3.38	1.93E-02
2	*CBLB*	4.54	1.74E-03	3	*CDH11*	5.94	1.10E-03
2	*COL1A1*	3.42	3.88E-02	3	*CREBBP*	3.94	4.65E-03
2	*CREB1*	3.75	1.79E-02	3	*DNM2*	−3.27	4.73E-02
2	*ERBB2*	3.96	1.39E-02	3	*EML4*	−3.26	1.87E-02
2	*ERBB4*	−5.98	4.53E-08	3	*ERCC4*	−4.05	2.27E-03
2	*ERCC4*	−5.50	1.37E-05	3	*FGFR1*	−2.98	4.97E-02
2	*FGFR1*	−4.56	1.17E-02	3	*FSTL3*	4.23	5.35E-03
2	*FGFR1*	−6.44	4.06E-02	3	*GOLGA5*	−2.82	4.28E-02
2	*FLT3*	−4.82	3.02E-03	3	*HERPUD1*	−3.21	1.67E-02
2	*FLT3*	−4.94	2.17E-03	3	*IL7R*	−4.38	2.09E-03
2	*FOXP1*	−3.23	3.85E-02	3	*KRAS*	4.35	1.61E-03
2	*FUS*	3.89	1.16E-02	3	*MAML2*	3.47	3.65E-02
2	*GNAS*	−3.81	5.77E-03	3	*MDM4*	−3.22	2.03E-02
2	*GSK3B*	3.33	1.60E-03	3	*MLH1*	4.12	6.01E-03
2	*HERPUD1*	−4.86	1.40E-04	3	*MLL3*	2.99	4.69E-02
2	*KTN1*	−3.84	5.91E-03	3	*MLL3*	−3.13	1.86E-02
2	*MAML2*	3.45	4.62E-02	3	*MLLT6*	3.08	3.75E-02
2	*MLL3*	3.46	1.77E-02	3	*NDRG1*	4.05	3.35E-03
2	*MLL3*	−3.47	6.01E-03	3	*NFIIB*	−3.10	3.29E-02
2	*NDRG1*	5.71	2.73E-05	3	*NOTCH2*	4.12	7.57E-03
2	*NFIIB*	−3.23	2.54E-02	3	*PALB2*	4.41	5.20E-03
2	*NFIIB*	−3.43	1.17E-02	3	*PICALM*	3.28	3.40E-02
2	*PIM1*	4.90	1.42E-03	3	*PIM1*	3.84	2.57E-02
2	*PPARG*	3.82	3.95E-02	3	*PPARG*	4.44	2.54E-02
2	*PRDM1*	4.36	3.80E-03	3	*REG4*	6.92	2.04E-07
2	*PRDM1*	3.66	2.19E-02	3	*REG4*	6.49	5.05E-07
2	*REG4*	4.57	1.85E-03	3	*REG4*	4.77	3.84E-04
2	*REG4*	3.43	1.76E-02	3	*RUNX1*	3.61	8.25E-03
2	*RUNX1*	3.92	6.18E-03	3	*TOP2A*	8.99	2.97E-04
2	*SET*	4.04	6.85E-03	3	*TOP2A*	8.30	1.24E-03
2	*SOX2*	3.57	2.75E-02	3	*TPM4*	3.99	8.54E-03
2	*TFPT*	3.87	1.21E-02				
2	*TFRC*	4.71	6.57E-04				
2	*TOP2A*	9.15	2.05E-04				
2	*TP53*	4.24	5.10E-03				
2	*TPM4*	4.24	3.63E-03				
2	*ZNF384*	−5.56	1.57E-05				

a Selected genes are genes that are reported in COSMIC.

b RNAseq was performed on patients 2 and 3.

Transcriptomic analysis led to the identification of significantly altered expression of genes that have been previously implicated in cancer. Significantly up-regulated genes in the tumor include *GSK3β* (glycogen synthase kinase 3 beta), *BRCA2*, *TP53*, *TOP2A* (topoisomerase II alpha 170 kDa), *BCL3* (B-cell CLL/lymphoma 3), and *REG4* (regenerating islet-derived family, member 4). Mutations in *GSK3β* have not been reported in PAC, but its increased expression in patient 2 may have a role in contributing to tumor malignancy and proliferation through SEMA3A [71]. *TOP2A* and *REG4* have been previously found to be associated with pancreatic cancer [70], [71], whereas *BCL3* has been found to be associated with other cancers [72], [73]. Additional up-regulated genes that fall in the COSMIC database include *MLL3* (myeloid/lymphoid or mixed-lineage leukemia 3), *BIRC3* (baculoviral IAP repeat containing 3), *ERBB2/HER2* (v-erb-b2 erythroblastic leukemia viral oncogene homolog 2), *PPARG* (peroxisome proliferator-activated receptor gamma), and *CBLB*, for which we also identified a copy number change. Mutations in *MLL3* have been previously identified in pancreatic cancer [72], [73], and *MLL3* was also identified as a candidate pancreatic cancer gene using a mutagenic screen in mice [74]. BIRC3, which acts to block apoptosis, was also previously reported to show increased expression in pancreatic cancer [75] and was found to be amplified in 22 pancreatic cancer cell lines [76]. *ERBB2/HER2*, an EGFR family tyrosine kinase that is involved in cell proliferation, has been frequently reported as demonstrating increased expression in pancreatic cancer [77], [78]. *PPARG* over-expression has also been previously identified in PAC and its over-expression was also found to be correlated with shorter survival [79]. Interestingly, inhibition of PPARG has been shown to block liver metastasis in a xenograft mouse model and motility of pancreatic cancer cells in vitro [80], and may thus represent a therapeutic target.

Down-regulated genes include *ERBB4* (v-erb-a erythroblastic leukemia viral oncogene homolog 4), *ERCC4* (excision repair cross-complementing rodent repair deficiency, complementation group 4), and *FGFR1* (fibroblast growth factor receptor 1). *ERCC4* has been reported to possibly be associated with risk of developing PAC [74], whereas *FGFR1* has been implicated in lung cancer [75], [76] and bladder carcinoma [77]. Decreased expression of *ERBB4* has been found in non-metastatic pancreatic cancer [81] and was reported to potentially influence metastasis of pancreatic cancer cells [82].

Of the fusion transcripts identified in patient 2, 2 genes that were identified as part of fusions also demonstrated statistically significant expression changes (q-value<0.05, corrected; Table S2). These genes include *LMO2* (LIM domain only 2 (rhombotin-like 1)) and *BACH1* (BTB and CNC homology 1, basic leucine zipper transcription factor 1), which were both identified in 1 putative fusion each. *LMO2* was identified as the 5′ gene in an interchromosomal fusion with *ACVR2A* (activin A receptor, type IIA). Interestingly, *LMO2* has been implicated in B-cell lymphoma [83] and prostate cancer [84] and is proposed to be a prognostic marker of longer survival in pancreatic cancer based on expression and immunohistochemical analyses [85]. Furthermore, a mutagenic screen aimed at identifying candidate pancreatic cancer genes led to the identification of point mutations in *ACVR2A*, in addition to other genes [74]. While only 2 non-junction-spanning reads support this chimera, this transcript may have relevant implications in patient 2's disease. *BACH1* was identified as the 3′ gene in an intrachromosomal fusion with *C21Orf109* (LINC00189; long intergenic non-protein coding RNA 189). 18 reads spanned the fusion junction to demonstrate increased confidence in this fusion. *BACH1* has been found to bind and inhibit TP53 such that its increased expression [86] and potential transcript fusion in patient 2 may influence tumor suppressor functions of TP53. While the exact function of *C21orf109* is unknown, long non-coding RNAs are known for their roles in transcriptional regulation. The putative chimera reported here may thus affect normal functions of this transcript and of *BACH1*. 2 additional predicted fusions were identified (*FAM18B2-CDRT4* and *SLC35A3-HIAT1*) with 17 and 15 reads spanning the junctions, respectively, but none of these genes have been reported in PAC or other cancers.

#### Summary

Following resection of the tumor, patient 2 was treated with chemoradiation followed by gemcitabine and erlotinib, and at 16 months post-resection, has not experienced a recurrence. The absence of somatic events affecting DNA repair genes and genes including *BRCA2*, *TP53*, *CDKN2A*, *SMAD4*, and *MYC*, as well as increased expression of *BRCA2* and *TP53* in the tumor, may all contribute to the status of this patient. If a recurrence were to occur, the increased expression of *TOP2A* indicates that topoisomerase inhibitors may be a possible treatment option. While additional studies are needed, up-regulated expression of *BIRC3* may provide evidence that sorafenib, a small molecule inhibitor of tyrosine and RAF kinases, and TRAIL (tumor necrosis factor-related apoptosis inducing ligand) may represent possible therapeutic options. Ricci *et al.* found that sorafenib down-regulates *BIRC3* and *MCL1* (myeloid cell leukemia sequence 1) expression and in doing so, causes TRAIL-resistant colon cancer cells to become sensitive to TRAIL, which promotes apoptosis [87] (we did not however identify statistically significant expression changes for *MCL1* and *TRAIL*). Lastly, the identification of over-expression of *ERBB2/HER2* provides evidence that trastuzumab, a monoclonal antibody that interferes with signaling through ERBB2/HER2, and/or lapatinib, which blocks ERBB1/EGFR and ERBB2 to obstruct cell growth and division, may be possible treatment options. The combined use of cetuximab and trastuzumab was found to be more beneficial than gemcitabine with regards to regression and survival when treating human pancreatic cancer xenografts [88]. Another study showed that a combined treatment of trastuzumab and matuzumab (anti-EGFR monoclonal antibody) on human pancreatic cancer xenografts demonstrated therapeutic benefit [89], whereas the use of multiple anti-ERBB2 antibodies targeting different ERBB2 epitopes also showed therapeutic benefit in mice [90].

### Patient 3 analysis

#### Whole genome analysis

Patient 3 did not harbor any events in *BRCA2*, *TP53*, *CDKN2A*, *SMAD4*, or *MYC*. However, patient 3 demonstrated a *KRAS* mutation for which glycine (G) is converted to arginine (R) at amino acid position 12 and also showed a somatic CNV gain of 1.38 (log2 scale) in *KRAS*. The missense G12R mutation has been reported in pancreatic cancer [91], [92] and other cancers [30], [93]. Outside of *KRAS*, we identified 13 additional SNVs and indels, of which 7 are predicted to be damaging or potentially damaging (Table 3).

*FUZ* (fuzzy homolog (Drosophila)), *KIAA1305* (NYNRIN; NYN domain and retroviral integrase containing), and *KIAA1737* (uncharacterized) have not been implicated in any cancers. *CA10* has been reported in chondroblastoma [94] and was identified as a putative methylation marker in bladder cancer [95], but has not been reported in PAC. *ZNF331* (zinc finger protein 331) may have a role in follicular thyroid adenomas [96], and has also been implicated as a potential tumor suppressor in gastric cancer [97]. Because the SNV identified in *ZNF331* is predicted to be damaging, its putative role as a tumor suppressor may represent a key event in this patient. Lastly, mutations in *LRRC7* (leucine rich repeat containing 7) have been identified in multiple cancers, including skin, ovarian, and breast cancer, but not in PAC.

Overall, CNV analysis of patient 3 led to the identification of 10 regions, covering 34 genes that demonstrated CNV alterations (COSMIC genes falling within these regions are listed in Table 4). Aside from a gain in *KRAS*, other key affected genes include *NOTCH2* (notch homolog 2), which also showed CNV gains, *PDE4DIP* (phosphodiesterase 4D interacting protein; myomegalin), and *FGFR3* (fibroblast growth factor receptor 3) and *MLLT4/AF6* (myeloid/lymphoid or mixed-lineage leukemia (trithorax homolog, Drosophila); translocated to, 4), which both showed CNV losses. Interestingly, one animal study showed that *KRAS*(G12D)/*NOTCH2* knockout mice survived longer and demonstrated no progression of pancreatic intraepithelial neoplasms (PanINs) compared to *KRAS*(G12D) and *KRAS* (G12D)/*NOTCH1* knockout mice, thereby, showing that NOTCH2 may have a significant role in tumor malignancy and development [98]. *PDE4DIP* and *FGFR3* have not been reported in PAC but *PDE4DIP* was identified as a tumor marker for esophageal squamous cell carcinoma [99], and mutations in *FGFR3* have been found in pancreatic endocrine tumors [100] as well as bladder cancer [101], [102]. Lastly, *MLLT4* has also not been reported in PAC but down-regulated expression of this gene is reported to be associated with increased likelihood of relapse in 14.5 to 15% of breast carcinoma cases as well as unfavorable prognosis [103], [104]. As previously mentioned, no significant somatic structural variants were identified for patient 3's tumor.

#### Whole transcriptome analysis

In patient 3, 1,939 genes were found to demonstrate significant expression changes (q<0.05, corrected for multiple testing) in the tumor. Selected genes are listed in Table 5 and genes that demonstrated both copy number changes and significant expression changes are listed in Table S1. Fusion transcripts identified in patient 3 are listed in Table S2. Like patient 2, somatic translocations were not identified so the fusion transcripts detected in patient 3 do not directly correlate with the tumor genome sequence.

Similar to patient 2, significant up-regulated expression was identified for *TOP2A*, *BCL3*, *BIRC3*, *MLL3*, *PPARG*, and *REG4*, and down-regulated expression was identified for *ERBB4*, *ERCC4*, and *FGFR1*. Patient 3's biopsy also demonstrated increased expression of *PTCH1* (patched 1), *BRCA1* (breast cancer 1, early onset), *DNM2* (dynamin 2), *MDM4* (p53 binding protein homolog (mouse)), *NOTCH2*, and *KRAS*. Although mutations in *PTCH1*, a tumor suppressor, have not been reported in PAC, its increased expression may influence tumor proliferation through the Sonic hedgehog pathway [105]. Up-regulated *BRCA1* expression suggests that patient 3's tumor may boast increased genomic stability; such an increase in expression has also been identified in putative tumor-initiating cells isolated from multiple pancreatic cancer cell lines compared to bulk cells [106]. Interestingly, up-regulated expression of *DNM2* has been reported in pancreatic cancer and was shown to be associated with increased tumor cell migration and invasion in human pancreatic cancer cells in vitro [107], and may thus represent a new therapeutic target for PAC. MDM4 normally acts to block TP53's tumor suppressor functions such that its increased expression in patient 3's tumor may be a key malignant event in this patient. Increased expression of *MDM4* has been identified in a number of other cancers that have wild type p53, including head and neck squamous carcinoma [108], breast cancer, and lymphoblastic leukemia [109]. One study described *MDM4* as an oncogene upon identifying the development of spontaneous tumors in conditional transgenic mice overexpressing *MDM4*, along with an increase in tumorigenesis in offspring when these mice were crossed with *TP53*+/− mice [110]. While no mutations were identified in *TP53* for this patient, up-regulated expression of *MDM4* and the other genes described here provide valuable information for the identification of new therapeutic targets and also provide insight on the biological processes that are occurring within the tumor.

In patient 3, we detected putative fusion transcripts supported by the identification of reads spanning the transcript breakpoint (Table S2). With the exception of *CAV1* (caveolin-1), the genes identified in these fusions have not been reported in PAC. Over expression of *CAV1*, which also demonstrated increased expression (q<0.05, corrected) in our study, has been found to be associated with disease recurrence in pancreatic cancer patients [111]. Its increased expression and potential role in an interchromosomal fusion transcript in our results indicates that these events may influence tumor progression in this patient. Additional fusions, that did not harbor junction-spanning reads, but have been previously reported in PAC, were also identified. *BCL3*, which we've previously described in this patient, was detected as the 3′ gene in a fusion with *PHLPPL* (PH domain and leucine rich repeat protein phosphatase 2). Another gene that was identified in our transcriptomic analyses in this patient and that was found to be a fusion gene is *REG4*, for which multiple fusions were predicted. These fusions include *REG4-SLC23A2* (solute carrier family 23 (nucleobase transporters), member 2) and *REG4-LARP1* (La ribonucleoprotein domain family, member 1). Although the 3′ genes in these fusions have not been reported in PAC, an *in vitro* study showed that LARP1 may have a key role in cell migration [112]. Other genes of interest that were found in separate putative fusions include *MAP4K4* (mitogen-activated protein kinase kinase kinase kinase 4), S100A4 (S100 calcium binding protein A4), *MMP7* (matrix metallopeptidase 7), and *IER3/IEX1* (immediate early response 3). *MAP4K4* was detected as the 3′ gene in a fusion with *APLP2* (amyloid beta (A4) precursor-like protein 2), which codes for a protein that was found in pancreatic cancer cell line supernatant [113]. While the effect of this putative fusion is unclear, *MAP4K4* over expression, which was also identified here, was reported in stage II PAC patients and was found to be correlated with negative prognosis in these patients [114]. *S100A4* was found to be in predicted intrachromosomal fusion with *LZIC* (leucine zipper and CTNNBIP1 domain containing). One study showed a relationship between S100A4 inhibition and increased gemcitabine sensitivity in PAC cell lines [115]. While implications for the predicted *S100A4-LZIC* fusion are not known, the increased expression of *S100A4* that we identified in our RNAseq analyses indicates that this gene may be a relevant therapeutic target.

We also identified *MMP7*, which demonstrated significant increased expression in patient 3's tumor, as the 3′ gene in a putative fusion with *EPHX1* (epoxide hydrolase 1, microsomal), which was shown to not play a role in pancreatic cancer [116]. Over expression of *MMP7*, which has roles in cell proliferation and differentiation, has been reported to be correlated with poor prognosis in PAC and tumor stage [117], [118], [119] and has also been reported specifically in liver metastases of pancreatic cancer [117]. Given these findings, the possible presence of the *MMP7* fusion transcript may not significantly affect *MMP7*'s normal functions given the diagnosis and outcome of patient 3. Another predicted chimera was an *IER3-SERPINA6* (serpin peptidase inhibitor, clade A (alpha-1 antiproteinase, antitrypsin), member 6) fusion—both genes in this fusion also showed statistically significant over expression in the tumor. While *SERPINA6* has not been reported as having a role in PAC, studies have shown that *IER3* expression is linked to both poor prognosis [120] and improved prognosis [121] in PAC patients. While additional experiments are necessary for clarifying the discrepancy across these findings, the presence of an *IER3* fusion may have had implications on this patient's prognosis. Because the effect of the fusions detected here are unclear, additional sequencing and compilation of chimeric transcripts are needed so that we can begin to unveil the role of these species on pancreatic tumorigenesis.

#### Summary

Prior to biopsy, patient 3 was first treated with TH-302, an investigational drug that activates nitroazole under hypoxic conditions plus gemcitabine as part of a phase I clinical trial. He had transient clinical benefit at first but progressed and was then treated with gemcitabine and nab-paclitaxel, but the disease continued to progress. Our identification of a copy number gain in and increased expression of *NOTCH2* may provide some explanation for the patient's responses to his first two treatments. Up-regulated expression of *NOTCH2* was identified in gemcitabine-resistant pancreatic cancer cells to suggest its possible involvement in chemotherapy resistance [122]. Increased expression of *NOTCH2* in patient 3 may be associated with disease progression following gemcitabine treatments. NOTCH2 inhibitors are thus a possible therapeutic option for this patient. A trial is currently recruiting stage IV pancreatic cancer patients, for whom tumor resection is not an option, to evaluate the efficacy of a combined therapy of MK0752, a NOTCH inhibitor, and gemcitabine hydrochloride. While additional analyses are needed, increased expression of *S100A4* in patient 3 also suggests that this may be key target as *S100A4* inhibition may be associated with increased sensitivity to gemcitabine. Another potential treatment is topoisomerase inhibitors given up-regulated *TOP2A* expression in the tumor. Similar to patient 2 and although additional studies are required, sorafenib and TRAIL may represent future options for patients whose tumors over express *BIRC3*. Lastly, increased expression of *MDM4* in patient 3's tumor indicates that MDM4 inhibitors may also be a possible future option for patients. This option is preceded by Wang *et al.*, who identified a benzofuroxan derivative that acts as an MDM4 inhibitor and showed that this small molecule inhibitor acts to promote apoptosis in a breast cancer cell line [123].

### Pathway Analysis

While the goal of this study is to perform patient-specific analyses, we also performed pathway analysis across all patients to evaluate affected biological processes. This type of analysis is preceded by Jones *et al.* who performed whole genome and expression analyses on 24 pancreatic ductal adenocarcinoma cell lines and xenografts [72]. In this study, mutations, copy number changes, deletions, and expression changes were identified using targeted sequencing of exons, microarrays, and mRNA sequencing using SAGE (Serial Analysis of Gene Expression) tags. Using this approach, the authors identified 12 core signaling pathways for pancreatic cancer. For our analyses, results from WGS were integrated with RNAseq data to identify pathways that may be affected across all 3 patients. The 142 identified genomic events, including all genes falling in regions demonstrating CNVs, were evaluated alongside significant expression changes (q<0.05, corrected) in patients 2 and 3.

**Figure 4:**
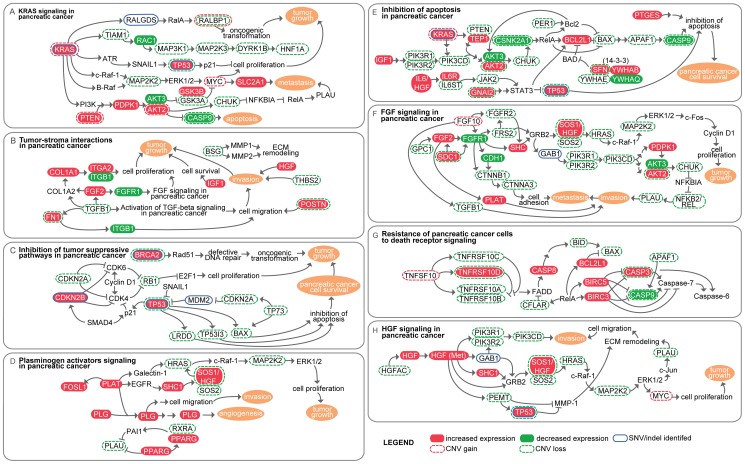
Pathway analysis of WGS and RNAseq results. Whole genome and RNAseq data were integrated and analyzed using GeneGo's Metaminer Pancreatic Cancer Disease module to identify pathways that may be affected by mutations and/or significant expression changes (q-value<0.05, corrected). The top pathways (minimum mapping p-value across all WGS and RNAseq datasets <0.05) are summarized based off of GeneGo maps. Breakdown of affected pathways in each patient are shown in Table S4.

Using GeneGo's Metaminer Pancreatic Cancer Disease module, we evaluated the extent to which 21 annotated pancreatic cancer pathways are affected in the three patients (Table S4). The top pathway maps (minimum mapping p-value<0.05) that demonstrated the lowest probability of genes mapping to the specified map by chance are summarized in Figure 4. Genes demonstrating both mutations and expression changes in the top maps are listed in Table S5. As expected, integrated analysis of WGS and RNAseq data indicated that the most highly affected pathway is KRAS signaling in pancreatic cancer. Affected genes include those that solely demonstrate mutations or expression changes, as well as those that demonstrate both mutations and expression changes. Known cancer genes that fall in the KRAS signaling pathway and that demonstrated alterations include *KRAS*, *TP53*, *MYC*, *PTEN* (phosphatase and tensin homolog), and *AKT2*.

Genomic events and expression changes were also analyzed across the entire GeneGo pathway database in order to perform an unbiased global analysis and to identify processes that may not be captured in the Pancreatic Cancer Disease module. The top map categories that were identified as demonstrating the largest number of alterations include prostatic neoplasms, hepatocellular carcinoma, and pancreatic neoplasms (Table S4 and Figure 4). Additional map categories outline hallmark processes of cancer and tumorigenic pathways. Identification of pathways that are implicated in other cancers (prostate and liver) provides insight as to possibly novel interactions that are not reported or less common in pancreatic cancer. Consistent with pathway analysis against MetaMiner's Pancreatic Cancer Disease Module, the most highly affected pathway was KRAS signaling in pancreatic cancer (Figure 4), followed by ligand-independent activation of androgen receptor. The latter map is annotated with cell progression, cell proliferation, and survival pathways in prostate cancer, and may provide clues into processes that may drive tumorigenesis in PAC.

As expected, processes in the top pathways (minimum mapping p-value<0.05) identified through NGS analyses overlap with the 12 core signaling pathways previously reported by Jones *et al.*, who also used GeneGo for pathway analysis. Overlapping processes and pathways include KRAS signaling, apoptosis, cell adhesion, and invasion. While apoptosis, cell adhesion, and invasion processes represent hallmark features of pancreatic cancer and other human cancers, the identification of KRAS signaling across multiple PAC samples, as well as the identification of previously reported KRAS mutations in the 3 patients analyzed here, emphasizes the tumorigenic role of KRAS signaling in pancreatic cancer. The high incidence of KRAS mutations in PAC [31], [124], along with the finding that patients who have a KRAS mutation have negative clinical outcomes when treated with a commonly prescribed combination of erlotinib, an EGFR inhibitor, and gemcitabine [125], further indicates that processes surrounding KRAS represent relevant therapeutic targets.

Although patient 1's tumor demonstrated the highest number of mutations, RNAseq data from patients 2 and 3 showed widespread pathway overlap with these genomic events. Patient 1 also uniquely harbors mutations that affect DNA repair pathways with respect to mismatch and nucleotide excision repair, DNA damage-induced responses, and BRCA1 as a transcription regulator. The larger number of identified genomic events in patient 1 may be associated with alterations in genes involved in DNA repair pathways and likely represents passenger mutations. Although tumors from patients 2 and 3 demonstrated fewer mutations, RNAseq data from these two patients suggest that common pathways are affected across all 3 patients.

Pathway analysis of WGS and RNAseq data allows us to understand which tumorigenic processes are present across the three patients. However, it is also important to recognize several caveats including: (1) mutations that are detected in larger genes (such as *MAP2K4*, *NCAM1*, *LAMA1*, and *LAMC1*, which are all over 100 kb) have a greater probability of representing a random mutation; the presence of such random events may bias pathway analysis; (2) alterations may influence additional key processes that are not annotated in the map database; (3) the tumor contents are 50% and 40 to 50% for patients 2 and 3, respectively, so that smaller, but potentially important, expression changes in tumor cells may not be readily identifiable; and (4) patient 2 was chemotherapeutically naïve and had her primary tumor sequenced whereas patients 1 and 3 were treated prior to biopsy collection and had their metastases sequenced. Despite these differences, pathway analysis allows us to evaluate commonly affected pathways across all 3 patients. *KRAS* is the only gene that harbored mutations (SNVs and a CNV) across all three patients and that also demonstrated a focal CNV gain and significant increased expression in patient 3.

## Conclusion

Due to the lack of effectiveness of current treatments for PAC patients, we are tasked with improving our understanding of genomic aberrations and processes that drive PAC tumorigenesis, tumor progression, and malignancy in order to identify and develop efficacious treatments. Our approach involves individually characterizing patients to fully understand the range of molecular events associated with this disease. In this study, we report our findings of 3 individual genomic characterizations of tumors collected from 3 separate patients. In 2 of the 3 patients, we additionally performed RNAseq on the same whole genome sequenced biopsies to identify significant expression changes and fusion transcripts that may be associated with tumorigenesis and that may be linked to the genomic events identified from WGS. With this patient-specific characterization, we identified potentially actionable therapeutic targets and contribute our findings to the research and clinical communities. Using this approach, we also detected aberrations that have not been previously reported in PAC, but may represent viable targets in other patients who also carry the same alteration. While further studies are needed to determine which aberrations are passenger and driver mutations, these results contribute valuable information to our understanding of the disease.

The utility of RNAseq data is clear when considering our analyses of patients 2 and 3, compared to patient 1. While WGS allowed us to identify non-synonymous mutations and copy number changes in patient 1, expression data provides more information on likely affected biological processes. As needle biopsies are most commonly performed, analyses are typically limited by the availability of tumor biopsy tissue. This limitation thereby obstructs proteomic analyses. However, by layering in RNAseq data, we acquire a more detailed picture of potentially tumorigenic events in individual patients. By evaluating these changes, our aim is to demonstrate the utility of using NGS to understand what molecular events are occurring in the tumors of separate patients and to move towards a more detailed understanding of the spectrum of aberrations that occur in this disease. In doing so, this information may point to additional therapeutic options that clinicians may consider during therapeutic selection. Furthermore, identification of targets that fall outside of FDA-approved pharmaceuticals or clinical trials serves to provide novel and relevant areas of research for drug development. One caveat here is that such analyses are dependent on the quality and tumor content of the biopsies that are collected. The percentage of tumor cells in the 3 analyzed patients' biopsies ranged from 40% to 60%, average mapped coverages ranged from 31× to 54× using WGS, and using RNAseq, over 100 million mapped reads were achieved in each of patients 2 and 3. We show that an average tumor content of approximately 50% is sufficient for NGS analysis of tumor biopsies. Under circumstances whereby only biopsies with lower tumor contents are available, NGS analyses may prove to be difficult, particularly for the identification of heterozygous mutations, and otherwise will require an increase in coverage and an increase in the number of reads needed to identify pertinent genomic events and changes in gene expression. A second caveat in this study is that mutations that were not present in the original tumor may arise while patients are undergoing therapy and potentially hinder the efficacy of the treatment. While our understanding of the details surrounding such events is limited, additional sequencing of patients at different time points before, during, and after treatments, will allow us to begin to understand the contribution of these aberrations to the disease.

Given our findings, the advantages of whole genome and transcriptome NGS in cancer patients are threefold—(1) foremost is our ability to survey the entire genome and transcriptome in order to detect abnormalities that may be missed using currently available cancer testing panels, (2) the identification of expression changes that may be associated with genomic events or that point to putative drug targets, and (3) the annotation of PAC genomes that provide insight into the molecular and cellular events involved in tumorigenesis. The utility of NGS has also been demonstrated in other sequencing studies that have used this technology to evaluate genomic rearrangements in pancreatic cancer [126] as well as differences in clonal populations between primary and metastatic pancreatic tumors [127]. Such advantages and applications are intertwined with rapid improvements in NGS technologies. The throughput for sequencing has nearly doubled within one year and is forecasted to continue to grow over the next few years. While turnaround time and the pipeline from sample collection to sequencing results are still being optimized, we demonstrate that NGS represents a compelling solution to obtaining detailed molecular information on tumor biopsies in order to provide guidance for therapeutic selection. Such an approach is applicable to all cancers for which tumor biopsy material can be acquired and is an obvious and powerful method for advancing our understanding of pancreatic cancer. Because we are still early in this process, the diversity of the findings in each of the 3 patients in this study does not come as a surprise. As we continue to sequence patients, we will acquire a better understanding of the compendium of events that have a role in the disease, determine what aberrations represent driver or passenger mutations, and strengthen our knowledge base for identifying and developing improved therapeutics.

## Supporting Information

Methods S1 Detailed methods are listed here.
(DOCX)

Table S1 **Selected genes demonstrating both CNVs and expression changes in patient tumors.** Selected genes that demonstrate a CNV gain or loss along with a significant (q-value<0.05) expression change are listed. ^a^RNAseq was performed for patients 2 and 3. ^b^Correlation between genomic event and expression change, e.g. + indicates a positive correlation between copy number change and expression change.
(DOCX)

Table S2 **ChimeraScan results for patients 2 and 3.** ChimeraScan was used to identify putative fusion transcripts based on RNAseq data collected from patients 2 and 3. Selected predicted fusions are listed along with significant expression changes where relevant.
(DOCX)

Table S3 **aCGH validation of CNVs identified using WGS.** To validate CNV alterations identified using whole genome sequencing (WGS), aCGH was performed on patient 1 and flow sorted aCGH was performed on patient 2. ^a^aCGH was performed on patient 1, flow sorted aCGH was performed on patient 2. ^b^P-values are calculated using the ADM2 algorithm [2] which generates ADM2 scores; p-values with a 0 value results when an interval has either a large copy number change, covers a large number of probes on the array, or both. The ADM2 score represents the deviation of the average of the normalized log ratios from its expected value of zero and is proportional to the height h (absolute average log ratio) of the genomic interval, and to the square root of the number of probes in the interval.
(DOCX)

Table S4 **Pathway analysis: Affected genes identified within each patient.** The total number of genes that fall in the specified pathway across WG and RNAseq datasets across all patients are shown along with the genes themselves and p-values associated with each patient for the specific pathway. ^a^Total number of objects/genes in pathway map. ^b^Number of genes demonstrating significant changes (q-value<0.05, corrected).
(DOCX)

Table S5 **Genes demonstrating mutations and expression changes in the top 10 pathways identified using GeneGo's Pancreatic Cancer Disease module.** Genes listed fall within the top ten pathways of GeneGo's Pancreatic Cancer Disease module. Genes show either a somatic alteration, significant expression change, or both.
(DOCX)
