# Supplementary material for: Genome-Wide Characterization of Pancreatic Adenocarcinoma Patients Using Next Generation Sequencing
Source: PLoS One. 2012 Oct 10;7(10):e43192. doi: 10.1371/journal.pone.0043192 (PMC3468610; doi:10.1371/journal.pone.0043192)
Supplement: Methods S1 — Detailed methods are listed here. (DOCX) [file pone.0043192.s001.docx]

**Genome-wide characterization of pancreatic adenocarcinoma patients using next generation sequencing**

**SUPPORTING INFORMATION**

**Supplementary Methods**

***Patient 1 history and sample acquisition***

A 55-year-old Caucasian man presented with poorly differentiated pancreatic adenocarcinoma in the body of the pancreas and associated liver metastases. He also had tumor-related deep venous thrombosis in the left lower extremity, multiple pulmonary emboli and diabetes as other disease related manifestations. He was started on systemic therapy with gemcitabine and nab-paclitaxel. He had a robust response to therapy with shrinkage of the primary pancreatic mass and liver lesions on computerized tomography (CT) scans, resolution of thrombi in his left lower extremity/pulmonary emboli, weight gain, improved appetite and decline in his carbohydrate antigen 19-9 (CA19-9) from 763 U/ml to 62 U/ml. Unfortunately, after 6 months of therapy he was found to have worsening in some liver metastases and a rise in the CA19-9 from 62 U/ml to 871 U/ml. An ultrasound-guided liver biopsy was performed which confirmed the presence of pancreatic adenocarcinoma. Biopsies were obtained under radiographic guidance of a responsive metastasis, non-responsive metastasis, and unaffected liver, flash frozen in liquid nitrogen, transported on dry ice, and then stored at -80 deg C until sample processing. The non-responsive metastasis was analyzed using WGS. At this point, the patient was empirically started on 5-fluorouracil/ leucovorin/oxaliplatin (FOLFOX) systemic therapy. Rapid tumor shrinkage within 2 months was noted once again and decline in CA19-9 to 26 U/ml was achieved [normal : <50 U/ml]. Six months after the initiation of FOLFOX, the patient developed abdominal discomfort, change in bowel habits, and nausea. A CT scan revealed new/worsening liver lesions, new pelvic adenopathy and mesenteric stranding despite a persistent normal CA19-9 value.

***Patient 2 history and sample acquisition***

A 76-year-old previously healthy woman was admitted through the emergency room with dizziness, abdominal discomfort, and diarrhea. A CT scan demonstrated a 3.3 x 2.8 cm hypodense solid mass with irregular borders in the tail of the pancreas. There were no enlarged lymph nodes and no evidence of metastatic disease. The serum CA19-9 level elevated to 615 U/ml (reference range < 36 U/ml).

The patient agreed to allow for donation of whole blood and tumor specimen into our IRB-approved pancreatic cancer biospecimens repository (NCI P01 Grant CA109552) [[1](#_ENREF_1)]. After induction of anesthesia but before the incision was made, 20 cc of whole blood was collected into EDTA containing tubes. Immediately after the tumor was removed, the surgeon harvested a 1 gm portion of tumor which was divided between tubes containing RNAlater™ and cryovials which were then placed into liquid nitrogen for rapid freezing.

The patient underwent an uncomplicated hand-assist laparoscopic en-bloc distal pancreatectomy and splenectomy to resect the tumor. Pathologic examination demonstrated an invasive moderately-differentiated adenocarcinoma of the pancreas measuring 4 cm. One of 18 regional lymph nodes was involved with cancer. The primary tumor extended to the peripancreatic adipose tissue, but surgical resection margins were free of tumor (R0 resection). Tumor staging by the AJCC 7th edition was stage IIB (pT3, N1, M0). Postoperatively, the patient did well. She received an adjuvant course of chemoradiation (4500 cGY at 180 cGy per fraction over 25 fractions) with intensity-modulated radiation therapy (IMRT) then a 6-month course of gemcitabine and erlotinib. There has been no sign of recurrence at follow-up examination 12 months after surgery.

***Patient 3 history and sample acquisition***

The patient is a 57-year-old man who was in a state of good health until sudden onset of abdominal symptoms with pain, nausea, and vomiting. On workup he was found to have elevated bilirubin and liver enzymes. Abdominal ultrasound showed multiple solid liver lesions and mild intrahepatic ductal dilatation on the right. He underwent a magnetic resonance cholangiopancreatography (MRCP) showing a 4.7 cm pancreatic head mass and liver lesions. Diffuse fatty hepatic infiltration was also noted. An ultrasound-guided liver biopsy was performed and pathology revealed metastatic adenocarcinoma of probable pancreatic origin. He then underwent endoscopic retrograde cholangiopancreatography (ERCP) with dual stent placement. Patient was diagnosed with stage IV adenocarcinoma of the pancreas with liver metastasis and signed a consent form for treatment protocols. Patient was initially treated on a phase I clinical trial combining an investigational drug and gemcitabine. He had an initial response after two cycles. However, after the third cycle, he experienced disease progression. Afterwards, he began gemcitabine and nab-paclitaxel (off protocol treatment). He tolerated the regimen fairly well and received two cycles. Unfortunately, the disease showed progression by CT scan. Then, he began FOLFOX chemotherapy, and after two cycles had a favorable response with a decrease in tumor markers and improvement in liver metastasis on CT scans. Tumor biopsies were obtained from a lesion in the medial segment of the left hepatic lobe at the periphery of the liver using a 17-gauge needle and CT fluoroscopic visualization.

***Sample assessment***

Three unique tumor samples were received for tumor confirmation, analyte (DNA) extraction and processing for downstream whole genome sequencing experiments. All tumor samples were obtained under institutional review protocols, were preserved as fresh frozen and reference DNA was obtained from peripheral blood mononuclear cells. Samples from patient 1 were frozen and received in three (3) portions of liver needle biopsy cores with direct confirmation of biopsy labeled ‘non-responsive’ as 60% tumor, biopsy labeled ‘responsive as 10% tumor and biopsy labeled ‘normal’ as normal hepatocytes. The second case (patient 2) was received fresh frozen and direct visualization showed 50% tumor and homogeneous pattern. The third case (patient 3) had a tumor content estimate of 40-50% tumor. Liver biopsies were obtained under radiographic guidance, flash frozen in liquid nitrogen and transported to TGen on dry ice and stored at -80 deg C until sample processing. Direct visualization of samples collected from all three patients were obtained by two-ink frozen quality control (QC) procedure to estimate tumor content and extent of tissue heterogeneity by a board certified pathologist (GH). Digital image files of all QC fragments were scanned Aperio GL scanner and image files stored on secure web-based viewing by Spectrum Plus (Aperio, Inc).

***Genomic DNA isolation***

*Fresh Frozen Tissue.* Tissue from the needle biopsy (patient 1) was disrupted and homogenized in Buffer RLT plus, Qiagen AllPrep DNA/RNA Mini Kit, using the Bullet Blender^TM^, Next Advance. Specifically, approximately 7 mg of tissue (patient 1) and ≤30 mg of tissue (patients 2 and 3) was transferred to a microcentrifuge tube containing 600 µl of Buffer RLT plus, and 9 mg of 1.6 mm stainless steel beads (patient 1) or 9 mg of 0.9 mm-2.0 mm RNase free stainless steel beads (patients 2 and 3). The tissue was homogenized in the Bullet Blender at room temperature at a Speed 10 for 5 minutes. The sample was centrifuged at full speed and the lysate was transferred to the Qiagen AllPrep DNA spin column. Genomic DNA purification was conducted as directed by the AllPrep DNA/RNA Mini Handbook, Qiagen. DNA was quantified using the Nanodrop spectrophotometer and quality was accessed from 260/280 and 260/230 absorbance ratios.

*Buffy Coat.* Blood leukocytes (buffy coat) were isolated from whole blood by centrifugation at 1500 x g for 10 minutes at room temperature. Cell pellets were resuspended in 600 µl Buffer RLT plus and homogenized in the Bullet Blender at room temperature at Speed 5 for 2 minutes. The homogenate was centrifuged at full speed and the supernatant was transferred to the Qiagen AllPrep DNA spin column. Genomic DNA purification was conducted as directed by the Qiagen AllPrep DNA/RNA Mini Handbook. DNA was quantified using the Nanodrop spectrophotometer and quality was accessed from 260/280 and 260/230 absorbance ratios.

***RNA Isolation (patients 2 and 3)***

*Fresh Frozen Tissue*. Tissue was disrupted and homogenized in Buffer RLT plus, Qiagen AllPrep DNA/RNA Mini Kit, using the Bullet BlenderTM, Next Advance. Specifically, tissue (~30 mg) was transferred to a microcentrifuge tube containing 600 ul of Buffer RLT plus, 9 mg of 0.9 mm-2.0 mm RNAse free stainless steel beads. The tissue was homogenized in the Bullet Blender at room temperature at Speed 10 for 5 minutes. The homogenate was centrifuged at full speed and the supernatant was transferred to the Qiagen AllPrep DNA spin column. Ethanol (70%) was added to the flow-through and the mixture was applied to an RNeasy spin column. Total RNA purification was conducted as directed by the AllPrep DNA/RNA Mini Handbook, Qiagen. RNA was quantified using the Nanodrop spectrophotometer and quality was assessed using the Agilent Bioanalyzer.

***Whole genome library preparation***

3 µg of genomic DNA from each sample (control and tumor) was used for library preparation. In summary, samples were fragmented using the Covaris S2 system (part#4387833) to a target fragment size of 300 to 350 base pairs (bp). Fragmentation was verified by running samples on a 2% TAE gel. Overhangs in the fragmented samples were then repaired to form blunt ends using T4 DNA polymerase and Klenow (New England Biolabs; NebNext DNA Sample Prep Master Mix Set I; catalog#E6040L), and products were cleaned using Agencourt Ampure magnetic beads (Beckman Coulter Genomics; catalog#A29153). Adenine bases were next ligated onto the blunted fragments using Klenow exo (NebNext DNA Sample Prep Master Mix Set I), and A-tailed products were cleaned using Ampure magnetic beads. Products were next quantified using Quant-iT Picogreen dsDNA reagent (Invitrogen; catalog#P11496) in triplicate with a 0 ng/µL to 200 ng/µL standard. To prepare for adaptor ligation, samples were vacuum dried to the appropriate volume to allow for a 10:1 adaptor to DNA molar ratio. Diluted paired end Illumina adapters were then ligated onto the A-tailed products using DNA ligase (NebNext DNA Sample Prep Master Mix Set I). Following ligation, samples were run on a 3% TAE gel at 120V for 2.5 hours to separate ligated products. X-tracta gel extractors (USA Scientific; catalog#5454-0100) were used to select ligation products at 300bp and 350bp for each sample. Ligated products were isolated from these gel punches using Freeze ‘N Squeeze DNA Gel Extraction Spin Columns (Bio-rad; catalog#732-6166), and cleaned using Ampure magnetic beads. 2X Phusion High-Fidelity PCR Master Mix (Finnzymes; catalog#F-531L) was used to perform PCR in quadruplicate (10uL ligation/reaction) to enrich for these products. Enriched PCR products were run on a 2% TAE gel and were selected using x-tracta gel extractors. PCR products were purified from gel punches using Freeze ‘N Squeeze DNA Gel Extraction Spin Columns. Extracted products were purified using Ampure magnetic beads and quantified using Agilent’s High Sensitivity DNA chip (catalog#5067-4626) on the Agilent 2100 Bioanalyzer (catalog#G2939AA). Following quantitation, samples were diluted to 10nM for sequencing.

***Whole transcriptome library preparation (patients 2 and 3)***

All RNA samples were analyzed on the Agilent Bioanalyzer RNA 6000 Nano Chip to validate RNA integrity (RIN ≥ 7.0). 10 ng of total RNA was used to generate whole transcriptome libraries for RNA sequencing. Using the Nugen Ovation RNA-Seq System (cat#7100-08), total RNA was used to generate double stranded cDNA, which was subsequently amplified using Nugen’s SPIA linear amplification process. Amplified products were cleaned using Qiagen’s QIAquick PCR Purification Kit (cat#28104) and quantitated using Quant-iT Picogreen. Amplified cDNA was input into Illumina’s TruSeq DNA Sample Preparation Kit – Set A (cat#FC-121-1001) for library preparation. In summary, 1 µg of amplified cDNA was fragmented on the Covaris E210 to a target insert size of 300bp, and end repaired and cleaned using Ampure XP beads. Samples were then adenylated at the 3’ end and indexed paired end adapters with T overhangs were ligated onto the A-tailed inserts. Ligation products were run on a 2% TAE gel and size selected at 400bp using x-tracta gel extractors (USA Scientific; cat#5454-0100). Ligation products were isolated from gel punches using Freeze ‘n Squeeze columns and Ampure XP beads. Cleaned ligation products were input into PCR to enrich for libraries. PCR products were cleaned using Ampure XP beads and quantified using the Agilent Bioanalyzer.

***PE next generation sequencing***

Tumor and normal libraries were prepared for whole genome and whole transcriptome paired end sequencing. Libraries were denatured using 2N NaOH and diluted with HT2 buffer (Illumina). 1% of denatured and diluted phiX was spiked into each lane to allow for error rate reporting on the HiSeq. Clusters were generated using Illumina’s cBot and HiSeq Paired End Cluster Generation Kits (catalog#PE-401-1001) and sequenced on Illumina’s HiSeq 2000 using Illumina’s HiSeq Sequencing Kit (catalog#FC-401-1001). For whole transcriptome sequencing, each library (tumor and normal) was run on separate lanes of a flowcell.

***Flow cytometry CGH for patient 2***

After hybridization, microarray slides were scanned using an Agilent 2565C DNA microarray scanner. Microarray images were analyzed using Agilent Feature Extraction software version 10.7 (FE 10.7) with default settings. All aCGH experiments were evaluated using a series of quality control (QC) metrics. These include background noise, signal intensities and signal to noise ratios for each dye-specific channel, the reproducibility of a series of replicate control probes on the arrays, and a measure of the spread of the distribution of the log2 ratios reported in each experiment. The data from arrays that pass the QC metrics were then analyzed in Genome Workbench using an aberration detection algorithm (ADM2) 47. Aberration calling thresholds for ADM2 are determined based on hybridizations with normal 46XY and 46XX DNA derived from control tissues. We use the distribution of log2 ratios for autosome probes and the corresponding chromosome X probes from control experiments to determine the error rates in our experiments and to select the statistical threshold for calling aberrant genomic intervals in the sorted samples. Copy number abnormalities (CNA) were calculated by three-probe and 0.2 log2 filters and an aberration detection module-1 algorithm [[2](#_ENREF_2)] with a threshold of 9.0. An interval-based text summary with all abnormalities was obtained and subsequently analyzed.

***Data analysis***

Raw sequence data in the form of .bcl files were generated by the Illumina HiSeq 2000. These data were converted to .qseq files using Illumina’s BCL Converter tool, and resulting .qseq files were used to generate .fastq files for downstream analysis. Fastq files were validated to evaluate the distribution of quality scores and to ensure that quality scores do not drastically drop over each read. Validated fastq files were aligned to the human reference genome (build 36) using the Burrows-Wheeler Alignment (bwa) tool [[3](#_ENREF_3)], which uses the Burrows-Wheeler Transform (BWT) algorithm. Following alignment, generated .sai files were used to create .sam (sequence alignment map) files by converting suffix array coordinates to chromosomal coordinates [[3](#_ENREF_3)]. Resulting .sam files were input into SAMtools [[4](#_ENREF_4)] to create binary sequence (.bam) files. PCR duplicates were flagged for removal using Picard (<http://picard.sourceforge.net>), and base quality scores were recalibrated using GATK (Genome Analysis Toolkit) [[5](#_ENREF_5)]. Mutation analysis was performed to identify SNPs, indels, and CNVs. Circos plots were generated for each patient to summarize results from all variant analyses [[6](#_ENREF_6)] (Supplementary Figures 1-3).

*Single nucleotide variant (SNV) identification*. SNP calling was performed using SolSNP (<http://sourceforge.net/projects/solsnp/files/SolSNP-1.01/>) and Mutation Walker, a tool developed in house and that incorporates variant discovery tools from GATK. SNPs that were called using both tools were compiled for further examination. Two sets of thresholds, strict and lenient, were enabled to reduce the false negative rate. Data from each of these two sets were visually examined for false positives to create a final filtered list of true SNVs, which were annotated with GENCODE using an internal annotation script.

SolSNP is an individual sample mutation detector implemented in Java. The algorithm is based on modified Kolmogorov-Smirnov like statistics, which incorporates base quality scores. The algorithm is non-parametric and makes no assumptions on the nature of the data. It compares the discrete sampled distribution, the pileup on each strand, to the expected distributions (according to ploidy). In the case of a diploid genome, both strands need to provide evidence for the variation. Zero quality bases are trimmed off the pileup before comparison.

While making somatic calls, SolSNP’s high quality genotype call is made for all callable loci of the normal sample. To reduce false negatives, variant loci in tumor samples are called with the Variant Consensus mode. Variant loci in tumor samples that exhibit a high quality homozygous reference genotype in the normal sample are considered as somatic. To call somatic variants, SolSNP is augmented by a Python script.

SIFT (Sorting Intolerant From Tolerant) or PolyPhen-2 (Polymorphism Phenotyping v2) was used to determine the effect of coding SNV’s on protein function for genes listed in Table 3 [[7](#_ENREF_7),[8](#_ENREF_8)].

*Insertion/deletion identification*. Indel calling was performed using GATK and a somatic indel detection tool developed in house. For detecting somatic indels we employed a two step strategy. In the first step we removed reads whose insert size lay outside the interval (50,500) from the tumor bam files. GATK was then used to generate a list of potential small indels from this bam. A customized Perl script, which uses the Bio-SamTools library from BioPerl [[9](#_ENREF_9)], takes these indel positions and for each of the indels, looks at the region in the normal sample that is 5 bases upstream from the start of the indel and 5 bases downstream from the end of the indel. An indel was determined to be somatic only if there was no indel detected in the region under consideration.

*Copy number analysis.* Copy number analysis was completed by determining the log2 difference of the the normalized physical coverage (or clonal coverage) for both germline and tumor samples separately across a sliding 2kb window of the mean, where clonal coverage was incremented for the length of the insert between the read pairs for insert sizes less than 3 standard deviations of the mean. High repeat regions such as centromeres were defined as where the log2 normalized coverage exceeded 3 in the germline sample and were thus excluded. Regions where the coverage was zero were replaced by 1 so that homozygous deletions avoid infinite values and are generally capped at approximately -3.

*Structural variant analysis.* CREST [[10](#_ENREF_10)] was used to identify translocations from sequence alignments of tumor and normal data by using Samtools [[3](#_ENREF_3)], BLAT [[11](#_ENREF_11)], cap3 [[12](#_ENREF_12)] assembly programs. Briefly, reference genome was converted to the 2-bit format using UCSC provided conversion utility faToTwoBit (http://hgdownload.cse.ucsc.edu/admin/exe/linux.x86_64/faToTwoBit). CREST was used to first identify soft-clipped alignments from the BWA generated tumor and normal BAM files and then to identify tumor specific structural variations. We also used SVDetect [[13](#_ENREF_13)] for translocation detection from discordant reads from the BAM files.

*RNAseq Analysis.* RNAseq data was aligned against human reference genome (build 36) with TopHat 1.2 [[14](#_ENREF_14)]. TopHat was run with option –G (--GTF) that allows the user to provide an annotation file that is used to build a known junctions reference. Human reference genome build 36 annotation GTF file was downloaded from Ensembl database (ftp://ftp.ensembl.org/pub/release-54/gtf/homo_sapiens/) for this purpose. RNAseq reads were only aligned against the autosomes and sex chromosomes. Mitochondrial DNA and annotations were removed from the genome and annotation references prior to alignment. Inner distance was calculated by subtracting read length and adapter from the total insert size. Default values were used for all other TopHat options.

Cuffdiff, a program that is part of the Cufflinks suite (v1.0.3) [[15](#_ENREF_15)], was used to identify differentially expressed genes and isoforms. A mask file was generated from the Ensembl GTF containing all rRNA and tRNA information to be ignored in the differential analysis. Differential analysis was performed on FPKM (Fragments Per Kilobase of transcript per Million fragments mapped) expression values calculated for gene and isoform. Each list (gene and isoform) was filtered for significantly (q-value<0.05) differentially expressed genes and sorted by ln(fold-change). Default values were used for all other Cuffdiff options for this analysis.

*Pathway analysis.* Integrative analysis of whole genome and transcriptomic data was performed using the Functional Ontology Enrichment Tool in MetaCore from GeneGo, Inc. (v6.8; Thomson Reuters Business, Philadelphia, PA). Pathway analysis specific to pancreatic cancer was performed using the MetaMiner (Oncology) Pancreatic Cancer Disease Module add-on. P-values associated with each analysis are calculated in MetaCore using a hypergeometric distribution. These significance values indicate the likelihood for which mapping of a gene to a specific pathway would occur by chance.

**References**

1. Demeure MJ, Sielaff T, Koep L, Prinz R, Moser AJ, et al. Multi-institutional tumor banking: lessons learned from a pancreatic cancer biospecimen repository. Pancreas 39: 949-954.

2. Lipson D, Aumann Y, Ben-Dor A, Linial N, Yakhini Z (2006) Efficient calculation of interval scores for DNA copy number data analysis. J Comput Biol 13: 215-228.

3. Li H, Durbin R (2009) Fast and accurate short read alignment with Burrows-Wheeler transform. Bioinformatics 25: 1754-1760.

4. Li H, Handsaker B, Wysoker A, Fennell T, Ruan J, et al. (2009) The Sequence Alignment/Map format and SAMtools. Bioinformatics 25: 2078-2079.

5. McKenna A, Hanna M, Banks E, Sivachenko A, Cibulskis K, et al. (2010) The Genome Analysis Toolkit: a MapReduce framework for analyzing next-generation DNA sequencing data. Genome Res 20: 1297-1303. Epub 2010 Jul 1219.

6. Krzywinski M, Schein J, Birol I, Connors J, Gascoyne R, et al. (2009) Circos: an information aesthetic for comparative genomics. Genome Res 19: 1639-1645.

7. Adzhubei IA, Schmidt S, Peshkin L, Ramensky VE, Gerasimova A, et al. A method and server for predicting damaging missense mutations. Nat Methods 7: 248-249.

8. Ng PC, Henikoff S (2003) SIFT: Predicting amino acid changes that affect protein function. Nucleic Acids Res 31: 3812-3814.

9. Stajich JE, Block D, Boulez K, Brenner SE, Chervitz SA, et al. (2002) The Bioperl toolkit: Perl modules for the life sciences. Genome Res 12: 1611-1618.

10. Wang J, Mullighan CG, Easton J, Roberts S, Heatley SL, et al. (2011) CREST maps somatic structural variation in cancer genomes with base-pair resolution. Nat Methods 8: 652-654. doi: 610.1038/nmeth.1628.

11. Kent WJ (2002) BLAT--the BLAST-like alignment tool. Genome Res 12: 656-664.

12. Huang X, Madan A (1999) CAP3: A DNA sequence assembly program. Genome Res 9: 868-877.

13. Zeitouni B, Boeva V, Janoueix-Lerosey I, Loeillet S, Legoix-ne P, et al. (2010) SVDetect: a tool to identify genomic structural variations from paired-end and mate-pair sequencing data. Bioinformatics 26: 1895-1896.

14. Trapnell C, Pachter L, Salzberg SL (2009) TopHat: discovering splice junctions with RNA-Seq. Bioinformatics 25: 1105-1111.

15. Trapnell C, Williams BA, Pertea G, Mortazavi A, Kwan G, et al. Transcript assembly and quantification by RNA-Seq reveals unannotated transcripts and isoform switching during cell differentiation. Nat Biotechnol 28: 511-515.
