# Supplementary material for: Genome-Wide Characterization of Pancreatic Adenocarcinoma Patients Using Next Generation Sequencing
Source: PLoS One. 2012 Oct 10;7(10):e43192. doi: 10.1371/journal.pone.0043192 (PMC3468610; doi:10.1371/journal.pone.0043192)
Supplement: Table S1 — Selected genes demonstrating both CNVs and expression changes in patient tumors. Selected genes that demonstrate a CNV gain or loss along with a significant (q-value<0.05) expression change are listed. aRNAseq was performed for patients 2 and 3. bCorrelation between genomic event and expression change, e.g. + indicates a positive correlation between copy number change and expression change. (DOCX) [file pone.0043192.s002.docx]

**Table S1. Selected genes demonstrating both CNVs and expression change in patient tumors^a^**

|  |  |  |  | Genomic event | | Expression change | |  |  |
| --- | --- | --- | --- | --- | --- | --- | --- | --- | --- |
| Chr | Location | Gene Name | Patient | Variant Type | Change associated with variant | Fold Change | p-value (uncorrected) | q-value (corrected) | Correlation |
| 1 | 120151400 | *REG4* | 3 | CNV-Gain | 1.8 | 6.92 | 6.00E-10 | 2.04E-07 | + |
| 1 | 120411200 | *NOTCH2* | 3 | CNV-Gain | 1.9 | 4.12 | 1.57E-04 | 7.57E-03 | + |
| 12 | 23989600 | *SOX5* | 3 | CNV-Gain | 1.7 | 3.95 | 2.83E-04 | 1.17E-02 | + |
| 12 | 25119000 | *LRMP* | 3 | CNV-Gain | 1.7 | -3.87 | 1.76E-04 | 8.20E-03 | - |
| 12 | 25266400 | *KRAS* | 3 | CNV-Gain | 1.4 | 4.35 | 2.08E-05 | 1.61E-03 | + |
| 1 | 61339600 | *NFIA* | 2 | CNV-Loss | -1.1 | -4.93 | 2.16E-06 | 3.42E-04 | + |
| 1 | 78763300 | *PTGFR* | 2 | CNV-Loss | -1.1 | -4.04 | 2.38E-04 | 1.12E-02 | + |
| 1 | 97732600 | *DPYD* | 2 | CNV-Loss | -1.1 | -4.34 | 5.41E-04 | 2.01E-02 | + |
| 1 | 198353700 | *NR5A2* | 2 | CNV-Loss | -1.2 | -7.73 | 2.43E-12 | 1.40E-09 | + |
| 3 | 106962500 | *CBLB* | 2 | CNV-Gain | 1.4 | 4.54 | 1.88E-05 | 1.74E-03 | + |
| 5 | 32117500 | *PDZD2* | 2 | CNV-Gain | 1.2 | 3.37 | 1.33E-03 | 3.80E-02 | + |
| 5 | 36651400 | *SLC1A3* | 2 | CNV-Gain | 1.2 | 4.10 | 1.64E-04 | 8.59E-03 | + |
| 5 | 41204800 | *C6* | 2 | CNV-Gain | 1.1 | -4.65 | 3.18E-06 | 4.61E-04 | - |
| 8 | 134351300 | *NDRG1* | 2 | CNV-Gain | 1.2 | 5.71 | 1.03E-07 | 2.73E-05 | + |
| 17 | 90700 | *RPH3AL* | 2 | CNV-Loss | -1.3 | -4.17 | 2.19E-04 | 1.05E-02 | + |
| 18 | 9543600 | *PPP4R1* | 2 | CNV-Gain | 1.2 | 3.91 | 1.68E-04 | 8.70E-03 | + |
| 18 | 11723600 | *GNAL* | 2 | CNV-Gain | 1.2 | 5.68 | 3.76E-04 | 1.52E-02 | + |
