# Supplementary material for: Genome-Wide Characterization of Pancreatic Adenocarcinoma Patients Using Next Generation Sequencing
Source: PLoS One. 2012 Oct 10;7(10):e43192. doi: 10.1371/journal.pone.0043192 (PMC3468610; doi:10.1371/journal.pone.0043192)
Supplement: Table S2 — ChimeraScan results for patients 2 and 3. ChimeraScan was used to identify putative fusion transcripts based on RNAseq data collected from patients 2 and 3. Selected predicted fusions are listed along with significant expression changes where relevant. (DOCX) [file pone.0043192.s003.docx]

**Table S2. ChimeraScan results for patients 2 and 3**

| **Patient** | **5p chr.** | **5p start location** | **5p end location** | **5p strand** | **5p gene** | **5p gene fold change** | **5p gene q-value** | **3p chr.** | **3p start location** | **3p end location** | **3p strand** | **3p gene** | **# total reads** | **# reads spanning breakpoint** | **3p gene fold change** | **3p gene q-value** |
| --- | --- | --- | --- | --- | --- | --- | --- | --- | --- | --- | --- | --- | --- | --- | --- | --- |
| 2 | 21 | 29487685 | 29488451 | + | C21orf109 |  |  | 21 | 29615412 | 29656085 | + | BACH1 | 19 | 18 | 3.15 | 3.54E-02 |
| 2 | 17 | 15389823 | 15407569 | - | FAM18B2,FAM18B |  |  | 17 | 15280062 | 15282241 | - | CDRT4 | 17 | 17 |  |  |
| 2 | 1 | 100208127 | 100253563 | + | SLC35A3 |  |  | 1 | 100288052 | 100321516 | + | HIAT1 | 15 | 15 |  |  |
| 2 | 10 | 50735357 | 51041336 | - | PARG |  |  | 10 | 51402777 | 51404615 | + | AK098044 | 4 | 4 |  |  |
| 2 | 19 | 63778638 | 63789534 | + | MGC2752 |  |  | 1 | 793319 | 793917 | - | BC047940 | 5 | 4 |  |  |
| 2 | 1 | 1589628 | 1591440 | - | LOC728661 |  |  | 1 | 1576685 | 1578810 | - | CDC2L1 | 2 | 2 |  |  |
| 2 | 1 | 1589628 | 1591440 | - | LOC728661 |  |  | 1 | 1560962 | 1644129 | - | CDC2L1 | 2 | 2 |  |  |
| 2 | 11 | 33836698 | 33843272 | - | LMO2 | 3.77 | 2.03E-02 | 2 | 148373496 | 148404862 | + | ACVR2A | 2 | 0 |  |  |
| 3 | 4 | 102336095 | 102487056 | - | PPP3CA |  |  | 4 | 52424359 | 52477759 | + | DCUN1D4 | 50 | 50 |  |  |
| 3 | 8 | 62756080 | 62789555 | - | ASPH | 4.87 | 2.15E-04 | 8 | 48788603 | 48811027 | + | KIAA0146 | 13 | 13 |  |  |
| 3 | 2 | 55313327 | 55315601 | + | RPS27A |  |  | 7 | 115953814 | 115988465 | + | CAV1 | 9 | 9 | 3.10 | 3.47E-02 |
| 3 | 6 | 43097362 | 43101856 | + | C6orf153 |  |  | 19 | 6628845 | 6653589 | - | C3 | 10 | 6 |  |  |
| 3 | 7 | 99771672 | 99774447 | + | PILRB |  |  | 7 | 99649565 | 99649945 | + | STAG3 | 18 | 6 |  |  |
| 3 | X | 12903145 | 12905266 | + | TMSB4X | 3.24 | 2.15E-02 | 3 | 99997503 | 100003182 | - | DCBLD2 | 3 | 3 | 4.63 | 4.85E-04 |
| 3 | 16 | 70273154 | 70307243 | - | PHLPPL | -4.98 | 3.24E-04 | 19 | 49953820 | 49955140 | + | BCL3 | 5 | 0 | 4.43 | 4.40E-03 |
| 3 | 16 | 67328695 | 67426944 | + | CDH1 | -3.38 | 1.93E-02 | 16 | 14437059 | 14437254 | - | PARN | 8 | 0 |  |  |
| 3 | 1 | 150271605 | 150276134 | - | S100A11 | 2.98 | 4.35E-02 | 1 | 559395 | 560211 | + | CR615613 | 8 | 0 |  |  |
| 3 | 1 | 224064453 | 224098946 | + | EPHX1 | 4.20 | 3.36E-03 | 11 | 101896448 | 101901004 | - | MMP7 | 5 | 0 | 5.43 | 3.08E-05 |
| 3 | 6 | 30818954 | 30820305 | - | IER3 | 4.36 | 1.23E-03 | 14 | 93840337 | 93840692 | - | SERPINA6 | 2 | 0 | 3.38 | 3.17E-02 |
| 3 | 1 | 151782718 | 151784905 | - | S100A4 | 5.82 | 5.13E-06 | 1 | 9912362 | 9913101 | - | LZIC | 3 | 0 |  |  |
| 3 | 1 | 120138230 | 120155725 | - | REG4 | 6.92 | 2.04E-07 | 20 | 4781001 | 4798698 | - | SLC23A2 | 2 | 0 | 3.85 | 7.36E-03 |
| 3 | 1 | 120138230 | 120155725 | - | REG4 | 6.92 | 2.04E-07 | 5 | 154153552 | 154177355 | + | LARP1 | 5 | 0 |  |  |
| 3 | 1 | 68716057 | 68720508 | - | DEPDC1-V1,DEPDC1 |  |  | 7 | 6382014 | 6410122 | + | RAC1 | 7 | 0 | -4.86 | 1.48E-04 |
| 3 | 19 | 59473215 | 59476210 | - | MIR,LILRB2 |  |  | X | 152427963 | 152428162 | + | BGN | 4 | 0 | 3.03 | 4.31E-02 |
| 3 | 1 | 152460067 | 152464312 | + | UBAP2L |  |  | 17 | 34126145 | 34139581 | + | MLLT6 | 7 | 0 | 3.08 | 3.75E-02 |
| 3 | 11 | 129445010 | 129496914 | + | APLP2 |  |  | 2 | 101806821 | 101877583 | + | MAP4K4 | 6 | 0 | 4.52 | 6.58E-04 |
| 3 | 12 | 52960754 | 52962715 | + | HNRNPA1 |  |  | 10 | 5250680 | 5250911 | + | AKR1C4 | 5 | 0 | 7.35 | 1.63E-07 |
