# Supplementary material for: Genome-Wide Characterization of Pancreatic Adenocarcinoma Patients Using Next Generation Sequencing
Source: PLoS One. 2012 Oct 10;7(10):e43192. doi: 10.1371/journal.pone.0043192 (PMC3468610; doi:10.1371/journal.pone.0043192)
Supplement: Table S3 — aCGH validation of CNVs identified using WGS. To validate CNV alterations identified using whole genome sequencing (WGS), aCGH was performed on patient 1 and flow sorted aCGH was performed on patient 2. aaCGH was performed on patient 1, flow sorted aCGH was performed on patient 2. bP-values are calculated using the ADM2 algorithm [2] which generates ADM2 scores; p-values with a 0 value results when an interval has either a large copy number change, covers a large number of probes on the array, or both. The ADM2 score represents the deviation of the average of the normalized log ratios from its expected value of zero and is proportional to the height h (absolute average log ratio) of the genomic interval, and to the square root of the number of probes in the interval. (DOCX) [file pone.0043192.s004.docx]

**Table S3. aCGH validation of CNVs identified using WGS**

|  |  |  |  | **WGS** |  | **aCGH^a^** |  |  |
| --- | --- | --- | --- | --- | --- | --- | --- | --- |
| **Patient** | **Gene** | **Chr** | **Location** | alteration | CNV | alteration | CNV | P-value^b^ |
| 1 | *BRCA2* | 13 | 31842500 | loss | -0.914 | loss | -0.745581 | 0 |
| 1 | *MYC* | 8 | 128817000 | gain | 0.827 | gain | 0.845799 | 2.94E-272 |
| 1 | *TP53* | 17 | 7514500 | loss | -1.017 | loss | -0.774881 | 0 |
| 1 | *MDM2* | 12 | 67512500 | loss | -1.02 | loss | -0.759555 | 0 |
| 1 | *APC* | 5 | 112119000 | loss | -0.967 | loss | -0.795793 | 0 |
| 1 | *MAP2K4* | 17 | 11865500 | loss | -0.913 | loss | -0.774881 | 0 |
| 1 | *AKT2* | 19 | 45459000 | loss | -1.019 | loss | -0.789383 | 0 |
| 1 | *RB1* | 13 | 47947500 | loss | -1.004 | loss | -0.745581 | 0 |
| 1 | *PIK3R1* | 5 | 67558000 | loss | -0.836 | loss | -0.795793 | 0 |
| 1 | *MLLT3* | 9 | 20613000 | loss | -2.649 | loss | -0.860752 | 0 |
| 1 | *FGFR2* | 10 | 123248500 | loss | -0.862 | loss | -0.75481 | 0 |
| 1 | *ALK* | 2 | 29407000 | loss | -0.909 | loss | -0.760499 | 0 |
| 1 | *EML4* | 2 | 42384000 | loss | -0.951 | loss | -0.760499 | 0 |
| 1 | *HRAS* | 11 | 522500 | loss | -1.403 | loss | -0.765473 | 0 |
| 1 | *FHIT* | 3 | 60402500 | loss | -0.988 | loss | -1.818803 | 3.808E-54 |
| 1 | *CDKN2A* | 9 | 21985000 | loss | -2.669 | loss | -2.483713 | 0 |
| 2 | *CBLB* | 3 | 106962500 | gain | 1.371 | gain | 1.271541 | 0 |
| 2 | *IL7R* | 5 | 35901300 | gain | 1.105 | gain | 0.835479 | 0 |
| 2 | *LIFR* | 5 | 38521600 | gain | 1.126 | gain | 0.835479 | 0 |
| 2 | *NDRG1* | 8 | 134319500 | gain | 0.817 | gain | 0.744292 | 0 |
