# Supplementary material for: Genome-Wide Characterization of Pancreatic Adenocarcinoma Patients Using Next Generation Sequencing
Source: PLoS One. 2012 Oct 10;7(10):e43192. doi: 10.1371/journal.pone.0043192 (PMC3468610; doi:10.1371/journal.pone.0043192)
Supplement: Table S4 — Pathway analysis: Affected genes identified within each patient. The total number of genes that fall in the specified pathway across WG and RNAseq datasets across all patients are shown along with the genes themselves and p-values associated with each patient for the specific pathway. aTotal number of objects/genes in pathway map. bNumber of genes demonstrating significant changes (q-value<0.05, corrected). (DOCX) [file pone.0043192.s005.docx]

**Table S4. Pathway analysis: Affected genes identified within each patient**

|  | | **Total # objects^a^** | **# Genomic events** | | | | | | |  |  | **RNAseq expression^b^** | | | |  |  |
| --- | --- | --- | --- | --- | --- | --- | --- | --- | --- | --- | --- | --- | --- | --- | --- | --- | --- |
| **Pancreatic cancer maps** | |  | **Patient 1 #events** | **Genes** | **Patient 1 Pvalue** | **Patient 2 #events** | **Patient 2 Pvalue** | **Genes** | **Patient 3 #events** | **Patient 3 Pvalue** | **Genes** | **Patient 2 #genes** | **Patient 2 Pvalue** | **Genes** | **Patient 3 #genes** | **Patient 3 Pvalue** | **Genes** |
| **1** | **K-RAS signaling in pancreatic cancer** | 43 | 18 | *CASP9, AKT2, RALBP1, BAX, CHUK, DYRK1B, GSK3A, HNF1A, MAP2K2, MAP2K3, MAP3K1, MYC, PLAU, PTEN, TIAM1, TP53, KRAS, RALGDS* | 2.64E-03 | 2 | 4.44E-02 | RALBP1, KRAS | 1 | 1.61E-01 | KRAS | 6 | 5.53E-02 | *RAC1, AKT3, CASP9, GSK3B, TP53, SLC2A1* | 5 | 4.01E-01 | *RAC1, CASP9, SLC2A1, KRAS, AKT2* |
| **2** | **Tumor-stroma interactions in pancreatic cancer** | 29 | 7 | *BSG, FN1, POSTN, STK36, SUFU, TGFB1, THBS2* | 4.97E-01 | 0 | 1.00E+00 |  | 0 | 1.00E+00 |  | 4 | 5.19E-01 | *FGFR1, COL1A1, IGF1, POSTN* | 9 | 2.96E-03 | *ITGB1, FGFR1, ITGA2, FGF2, POSTN, FN1, PTCH1, IGF1, HGF* |
| **3** | **Inhibition of tumor suppressive pathways in pancreatic cancer** | 20 | 10 | *TP73, BAX, CDKN2A, CDKN2B, LRDD, MDM2, RB1, TP53I3, BRCA2, TP53* | 3.58E-03 | 0 | 1.00E+00 |  | 1 | 7.97E-02 | TP73 | 3 | 3.32E-01 | *BRCA2, TP53, CDKN2B* | 1 | 9.26E-01 | *CDKN2B* |
| **4** | **Plasminogen activators signaling in pancreatic cancer** | 33 | 7 | *HRAS, MAP2K2, PLAU, PLAUR, SOS1, SOS2, TGFB1* | 8.65E-01 | 0 | 1.00E+00 |  | 1 | 1.00E+00 | RXRA | 3 | 6.74E-01 | *PPARG, SHC1, PLAUR* | 5 | 1.47E-02 | *PLAT, PPARG, HGF, FOSL1, PLG* |
| **5** | **Inhibition of apoptosis in pancreatic cancer** | 63 | 19 | *CSNK2A1, CASP9, GNAI2, SFN, AKT2, ALOX12,APAF1, BAX, CHUK,IL6ST, JAK2, PER1, PIK3CD, PIK3R1, PIK3R2, PTEN, YWHAE, TP53, KRAS* | 2.45E-01 | 1 | 3.67E-01 | KRAS | 1 | 2.04E-01 | KRAS | 12 | 1.74E-02 | *EGF, AKT3, CSNK2A1, YWHAQ, CASP9, YWHAB, ERBB2, TP53, IGF1, GNAI2, PTGES, SFN* | 10 | 3.36E-01 | *CASP9, YWHAQ, CSNK2A1, YWHAB, KRAS, IL6R, IGF1, AKT2, BCL2L1, SFN* |
| **6** | **FGF signaling in pancreatic cancer** | 53 | 20 | *SDC1, AKT2, CHUK, CTNNA3, CTNNB1, FGFR2, FRS2, GPC1, HRAS, MAP2K2, NFKB2, PIK3CD, PIK3R1, PIK3R2, PLAU, REL, SOS1, SOS2, TGFB1, GAB1* | 2.58E-02 | 1 | 3.08E-01 | FGF10 | 0 | 1.00E+00 |  | 4 | 4.64E-01 | *FGFR1, AKT3, SDC1, SHC1* | 6 | 2.77E-01 | *CDH1, FGFR1, FGF2, PLAT, SDC1, AKT2* |
| **7** | **Resistance of pancreatic cancer cells to death receptor signaling** | 30 | 11 | *CASP9, CASP3, TNFRSF10D, APAF1, BAX, BID, CFLAR, TNFRSF10A, TNFRSF10B, TNFRSF10C, TNFSF10* | 2.87E-02 | 0 | 1.00E+00 |  | 0 | 1.00E+00 |  | 3 | 3.98E-01 | *CASP9, CASP3, BIRC3* | 6 | 7.75E-02 | *CASP9, TNFRSF10D, CASP8, BCL2L1, BIRC3, BIRC5* |
| **8** | **HGF signaling in pancreatic cancer** | 33 | 12 | *HGFAC, HRAS, MAP2K2, MYC, PIK3CD, PIK3R1, PIK3R2, PLAU, SOS1, SOS2, TP53, GAB1* | 4.50E-02 | 0 | 1.00E+00 |  | 0 | 1.00E+00 |  | 2 | 7.71E-01 | *TP53, SHC1* | 1 | 6.46E-01 | *HGF* |
| **9** | Role of cell adhesion molecules in progression of pancreatic cancer | 54 | 18 | *COL4A4, CASP3, PTK2, FN1, COL4A3, CTNNA3, CTNNB1, HRAS, LAMA1, MAP2K2, MYC, SOS1, SOS2, ST8SIA4, TCF7, TCF7L2, KRAS, NCAM1* | 1.47E-01 | 2 | 3.02E-01 | LAMC1, KRAS | 1 | 1.64E-01 | KRAS | 6 | 4.44E-01 | *COL4A4, JUP, COL1A1, COL4A1, CASP3, SHC1* | 10 | 6.68E-02 | *COL4A4, ITGB1, CDH1, PTK2, ITGA2, FN1, COL4A1, KRAS, ITGA3, ITGB3* |
| **10** | Activation of TGF-beta signaling in pancreatic cancer | 28 | 6 | *ICAM1, MAP2K4, SOS1, SOS2, TGFB1, KRAS* | 7.96E-01 | 1 | 1.93E-01 | KRAS | 1 | 1.02E-01 | KRAS | 2 | 7.53E-01 | *CLDN4, SHC1* | 3 | 3.82E-01 | *DAB2, PDGFA, KRAS* |
| **11** | Suppression of TGF-beta signaling in pancreatic cancer | 32 | 9 | *CDKN2B, GADD45B, MAP2K3, MAP3K4, MYC, PLAU, SKI, TGFB1, KRAS* | 2.22E-01 | 1 | 2.06E-01 | KRAS | 1 | 1.09E-01 | KRAS | 2 | 7.88E-01 | *BGN, CDKN2B* | 5 | 2.38E-01 | *SMAD6, BGN, ETS1, KRAS, CDKN2B* |
| **12** | Hedgehog signaling in pancreatic cancer | 33 | 7 | *IGF2, IHH, MAP2K2, STK36, SUFU, TGFB1, KRAS* | 6.53E-01 | 1 | 2.25E-01 | KRAS | 1 | 1.19E-01 | KRAS | 2 | 8.32E-01 | *CTSB, IGF2* | 6 | 1.56E-01 | *CDH1, IGF2, CTSB, PTCH1, KRAS, BCL2L1* |
| **13** | Role of stellate cells in progression of pancreatic cancer | 65 | 15 | *COL3A1, POSTN, PTK2, FN1, AKT2, CHUK, CTGF, HRAS, MAP2K2, PIK3CD, PIK3R1, PIK3R2, SOS1, SOS2, TGFB1* | 6.81E-01 | 0 | 1.00E+00 |  | 0 | 1.00E+00 |  | 7 | 2.18E-01 | *AKT3, CCL2, COL1A1, COL3A1, CXCL2, SHC1, POSTN* | 12 | 1.46E-01 | *ITGB1, PTK2, PDGFA, FGF2, POSTN, FN1, ITGB3, AKT2, CXCL2, FGB, FGG, FGA* |
| **14** | Neuropeptide signaling in pancreatic cancer | 47 | 12 | *PLCB1, PRKCE, AVP, CCKBR, CHUK, GNA11, MAP2K2, MYC, NFKB2, NTS, PRKCH, REL* | 3.56E-01 | 0 | 1.00E+00 |  | 1 | 1.78E-01 | ITPR2 | 4 | 7.15E-01 | *PLCB1, CCL2, ITPR3, AGT* | 6 | 5.08E-01 | *ITPR1, PRKCE, AVPR1A, ITPR3, HGF, AGT* |
| **15** | IGF-1 signaling in pancreatic cancer | 39 | 12 | *PTK2, HRAS, IRS1, JAK2, MAP2K2, NUAK1, PIK3CD, PIK3R1, PIK3R2, PRKCZ, SOS1, SOS2* | 2.04E-01 | 1 | 2.49E-01 | PRKAA1 | 0 | 1.00E+00 |  | 3 | 4.66E-01 | *ATM, IGF1, SHC1* | 3 | 6.14E-01 | *PIK3R3, PTK2, IGF1* |
| **16** | Role of metalloproteases and heparanase in progression of pancreatic cancer | 35 | 9 | *COL4A4, OCLN, BSG, COL4A3, HRAS, MAP2K2, SOS1, SOS2, KDR* | 6.87E-01 | 0 | 1.00E+00 |  | 0 | 1.00E+00 |  | 6 | 2.09E-01 | *COL4A4, OCLN, SERPINA1, COL4A1, SHC1, MMP7* | 5 | 3.32E-01 | *COL4A4, TJP1, COL4A1, MMP7, SERPINA1* |
| **17** | EGFR family signaling in pancreatic cancer | 84 | 23 | *ERBB4, NFKBIB, AKT2, CCNE1, CHUK, HRAS, JAK2, MAP2K2, MAP2K4, MAP2K7, MAP3K10, MYC, NFKB2, PIK3CD, PIK3R1, PIK3R2, PLAU, RB1, REL, RHOA, SOS1, SOS2, VAV1* | 2.27E-01 | 0 | 1.00E+00 |  | 0 | 1.00E+00 |  | 6 | 6.16E-01 | *ERBB4, EGF, RAC1, AKT3, ERBB2, SHC1* | 9 | 3.82E-01 | *RAC1, ATF2, ERBB4, PAK1, CDK2, NFKBIB, AKT2, BCL2L1, BIRC5* |
| **18** | Regulation of VEGF signaling in pancreatic cancer | 30 | 7 | *FLT1, HRAS, MAP2K2, NRP2, SOS1, SOS2, KDR* | 6.36E-01 | 0 | 1.00E+00 |  | 0 | 1.00E+00 |  | 2 | 7.53E-01 | *NRP2, SHC1* | 2 | 3.82E-01 | *FIGF, VEGFC* |
| **19** | Inflammatory mechanisms of pancreatic cancerogenesis | 76 | 18 | *GNAI2, IRF1, IFNGR2, CCL20, CCR6, CEBPG, CHUK, FOSB, FOSL2, ICAM1, IL6ST, JAK2, JUNB, JUND, NFKB2, PLAU, REL, TNFRSF1B* | 8.28E-01 | 0 | 1.00E+00 |  | 0 | 1.00E+00 |  | 5 | 7.94E-01 | *EGF, CCL2, CXCR4, GNAI2, AGT* | 9 | 8.08E-01 | *ATF2, IFNGR1, IRF1, IFNGR2, IL32, IL6R, BCL2L1, FOSL1, AGT* |
| **20** | Metabolism in pancreatic cancer cells | 19 | 8 | *SLC2A2, GLUD1, GPX1, RPE, SLC2A4, SLC2A5, TALDO1, TKT* | 9.93E-01 | 0 | 1.00E+00 |  | 0 | 1.00E+00 |  | 4 | 8.90E-01 | *SLC2A2, RPIA, SLC2A3, SLC2A1* | 3 | 9.86E-01 | *SLC2A2, SLC2A3, SLC2A1* |
| **21** | Mechanism of gemcitabine action in pancreatic cancer | 25 | 8 | *DCTD, POLE, POLE2, RRM1, RRM2B, XRCC5, POLA1, TP53* | 9.99E-01 | 0 | 1.00E+00 |  | 0 | 1.00E+00 |  | 2 | 9.95E-01 | *POLA1, TP53* | 0 | 1.00E+00 |  |
| **Top 5 overall GeneGo maps** | |  |  |  | 0.00E+00 |  | 0.00E+00 |  |  | 0.00E+00 |  |  | 0.00E+00 |  |  | 0.00E+00 |  |
| **22** | K-RAS signaling in pancreatic cancer (see above) |  |  |  | 3.27E-06 |  | 1.90E-02 |  |  | 9.24E-02 |  |  | 3.60E-03 |  |  | 7.95E-02 |  |
| **23** | Ligand-independent activation of androgen receptor | 91 | 31 | *AKT2, CTNNB1, FGFR2, FRS2, FZD10, FZD3, FZD5, FZD6, FZD7, HRAS, IRS1, JAK2, KLK3, MAP2K2, MDM2, MYC, NCOA1, PIK3CD, PIK3R1, PIK3R2, PPP2CA, PPP2R2A, PPP2R2C, PPP2R5E, PTEN, SOS1, SOS2, TCF7, TCF7L2, KRAS, GAB1* | 5.27E-05 | 1 | 2.85E-01 | KRAS | 1 | 1.41E-01 | KRAS | 9 | 5.94E-04 | *PRLR, EGF, FGFR1, AKT3, PPP2R3A, GSK3B, ERBB2, IGF1, SHC1* | 11 | 2.41E-03 | *PIK3R3, FGFR1, SRD5A1, FGF2, KRAS, IGFBP3, IGF1, AKT2, AR, BCL2L1, ABL1* |
| **24** | Transport_Macropinocytosis regulation by growth factors | 67 | 22 | *AKT2, CDC42, PLCB1, ACTB, ARPC2, ARPC3, CTBP1, DSTN, HRAS, IRS1, JAK2, MAPK7, PDE3B, PFN1, PIK3R1, PRKAB1, SOS1, SOS2, TIAM1, VAV1, WASF2, KRAS* | 3.35E-04 | 2 | 3.70E-02 | PRKAA1, KRAS | 1 | 1.30E-01 | KRAS | 8 | 7.43E-02 | *EGF, RAC1, AKT3, PLCB1, SHC1, CDC42, ARPC1B, WIPF1* | 9 | 1.04E-02 | *RAC1, PAK1, LEPR, WIPF1, KRAS, AKT2, HGF, CDC42, ARPC1B* |
| **25** | Inhibition of apoptosis in pancreatic cancer (see above) |  |  |  | 5.17E-03 |  | 2.42E-01 |  |  | 1.18E-01 |  |  | 3.97E-04 |  |  | 4.04E-02 |  |
| **26** | Development_FGF-family signaling | 52 | 16 | *AKT2, FGF8, FGFR2, FGFR3, FRS2, HRAS, HSPG2, MAP2K2, PIK3CD, PIK3R1, PIK3R2, PRKCD, PRKCE, PTPN11, SOS1, SOS2, GAB1* | 1.86E-03 | 1 | 2.23E-01 | FGF10 | 2 | 5.68E-03 | FGFR3, ITPR2 | 6 | 8.21E-02 | *RAC1, FGFR1, AKT3, ITPR3, SHC1* | 8 | 2.48E-02 | *RAC1, ITPR1, PIK3R3, FGFR1, FGF2, PRKCE, ITPR3, AKT2* |
