# Supplementary material for: Genome-Wide Characterization of Pancreatic Adenocarcinoma Patients Using Next Generation Sequencing
Source: PLoS One. 2012 Oct 10;7(10):e43192. doi: 10.1371/journal.pone.0043192 (PMC3468610; doi:10.1371/journal.pone.0043192)
Supplement: Table S5 — Genes demonstrating mutations and expression changes in the top 10 pathways identified using GeneGo's Pancreatic Cancer Disease module. Genes listed fall within the top ten pathways of GeneGo's Pancreatic Cancer Disease module. Genes show either a somatic alteration, significant expression change, or both. (DOCX) [file pone.0043192.s006.docx]

**Table S5. Genes demonstrating mutations and expression changes in the top 10 pathways identified using GeneGo’s Pancreatic Cancer Disease module**

|  | **Genomic events** | | | **Expression changes** | | |
| --- | --- | --- | --- | --- | --- | --- |
| Gene | Patient | Event | Alteration | Patient | Fold change (ln) | q-value (corrected) |
| *KRAS* | 1 | SNV | G12V |  |  |  |
| *KRAS* | 2 | SNV | G12V |  |  |  |
| *KRAS* | 3 | SNV | G12R |  |  |  |
| *KRAS* | 3 | CNV | 1.379 |  |  |  |
| *KRAS* |  |  |  | 3 | 4.35 | 1.61E-03 |
| *AKT2* | 1 | CNV | -1.019 |  |  |  |
| *AKT2* |  |  |  | 3 | 4.42 | 4.52E-02 |
| *GNAI2* | 1 | CNV | -1.021 |  |  |  |
| *GNAI2* |  |  |  | 2 | 4.75 | 8.11E-04 |
| *TP53* | 1 | SNV | R248W |  |  |  |
| *TP53* |  |  |  | 2 | 4.24 | 5.10E-03 |
| *CSNK2A1* | 1 | CNV | -0.905 |  |  |  |
| *CSNK2A1* |  |  |  | 2 | -3.89 | 6.07E-03 |
| *CSNK2A1* |  |  |  | 3 | -3.08 | 3.68E-02 |
| *CASP9* | 1 | CNV | -0.904 |  |  |  |
| *CASP9* |  |  |  | 2 | -3.29 | 3.66E-02 |
| *CASP9* |  |  |  | 3 | -4.16 | 3.13E-03 |
| *ERBB4* | 1 | CNV | -0.945 |  |  |  |
| *ERBB4* |  |  |  | 2 | -5.98 | 1.39E-05 |
| *ERBB4* |  |  |  | 3 | -3.45 | 1.93E-02 |
| *SOS1/HGF* | 1 | CNV | -1.011 |  |  |  |
| *SOS1/HGF* |  |  |  | 3 | 4.86 | 1.54E-03 |
| *NFKBIB* | 1 | CNV | -1.057 |  |  |  |
| *NFKBIB* |  |  |  | 3 | 3.75 | 2.64E-02 |
| *COL4A4* | 1 | CNV | -0.858 |  |  |  |
| *COL4A4* |  |  |  | 2 | -5.11 | 3.20E-04 |
| *COL4A4* |  |  |  | 3 | -6.11 | 1.93E-04 |
| *PTK2* | 1 | CNV | 0.843 |  |  |  |
| *PTK2* |  |  |  | 3 | -3.33 | 2.94E-02 |
| *FN1* | 1 | CNV | -0.957 |  |  |  |
| *FN1* |  |  |  | 3 | 4.00 | 4.37E-03 |
| *POSTN* | 1 | CNV | -0.833 |  |  |  |
| *POSTN* |  |  |  | 2 | 5.63 | 2.66E-05 |
| *POSTN* |  |  |  | 2 | 5.61 | 7.65E-05 |
| *POSTN* |  |  |  | 2 | 4.10 | 1.56E-02 |
| *POSTN* |  |  |  | 3 | 3.84 | 2.59E-02 |
| *POSTN* |  |  |  | 3 | 3.31 | 4.97E-02 |
| *COL3A1* | 1 | CNV | -0.984 |  |  |  |
| *COL3A1* |  |  |  | 2 | 3.73 | 1.95E-02 |
